# T-Type Calcium Channel: A Privileged Gate for Calcium Entry and Control of Adrenal Steroidogenesis

**DOI:** 10.3389/fendo.2016.00043

**Published:** 2016-05-20

**Authors:** Michel F. Rossier

**Affiliations:** ^1^Service of Clinical Chemistry and Toxicology, Hospital of Valais, Sion, Switzerland; ^2^Department of Human Protein Science, Faculty of Medicine, University of Geneva, Geneva, Switzerland

**Keywords:** adrenal cortex, steroidogenesis, aldosterone, cortisol, ACTH, calcium signaling, electrophysiology, T-type calcium channels

## Abstract

Intracellular calcium plays a crucial role in modulating a variety of functions such as muscle contraction, hormone secretion, gene expression, or cell growth. Calcium signaling has been however shown to be more complex than initially thought. Indeed, it is confined within cell microdomains, and different calcium channels are associated with different functions, as shown by various channelopathies. Sporadic mutations on voltage-operated L-type calcium channels in adrenal glomerulosa cells have been shown recently to be the second most prevalent genetic abnormalities present in human aldosterone-producing adenoma. The observed modification of the threshold of activation of the mutated channels not only provides an explanation for this gain of function but also reminds us on the importance of maintaining adequate electrophysiological characteristics to make channels able to exert specific cellular functions. Indeed, the contribution to steroid production of the various calcium channels expressed in adrenocortical cells is not equal, and the reason has been investigated for a long time. Given the very negative resting potential of these cells, and the small membrane depolarization induced by their physiological agonists, low threshold T-type calcium channels are particularly well suited for responding under these conditions and conveying calcium into the cell, at the right place for controlling steroidogenesis. In contrast, high threshold L-type channels are normally activated by much stronger cell depolarizations. The fact that dihydropyridine calcium antagonists, specific for L-type channels, are poorly efficient for reducing aldosterone secretion either *in vivo* or *in vitro*, strongly supports the view that these two types of channels differently affect steroid biosynthesis. Whether a similar analysis is transposable to fasciculata cells and cortisol secretion is one of the questions addressed in the present review. No similar mutations on L-type or T-type channels have been described yet to affect cortisol secretion or to be linked to the development of Cushing syndrome, but several evidences suggest that the function of T channels is also crucial in fasciculata cells. Putative molecular mechanisms and cellular structural organization making T channels a privileged entry for the “steroidogenic calcium” are also discussed.

## Introduction

Voltage-operated calcium channels play a crucial role in signal transduction of many excitable and non-excitable cell types (1). While a rapid modulation of their activity by hormone-stimulated kinases and/or G proteins has been recognized for a long time (2, 3), a control of their expression levels in the cell has been also described (4).

Among these channels, low threshold-activated, T-type (for transient and tiny current) calcium channels appear distinct concerning their electrophysiological and molecular properties. Although their existence has been shown early by studies of their voltage-dependence, kinetics, and single channel conductance (5–7), their cloning and their molecular characterization has been delayed, in part because of a lack of specific pharmacological tools and because they share less structural homology with the rest of the voltage-operated calcium channels. Thus, in the light of their molecular structure (8, 9), T-type calcium channels have been proposed to play specific physiological roles within the cells, and it has been suggested that they could be involved, depending on the levels of their expression, in the development of several diseases, such as hypertension, cardiac failure, epilepsy, or cancer (4).

### Electrophysiological Properties of T-Type Calcium Channels

**Figure 1:**
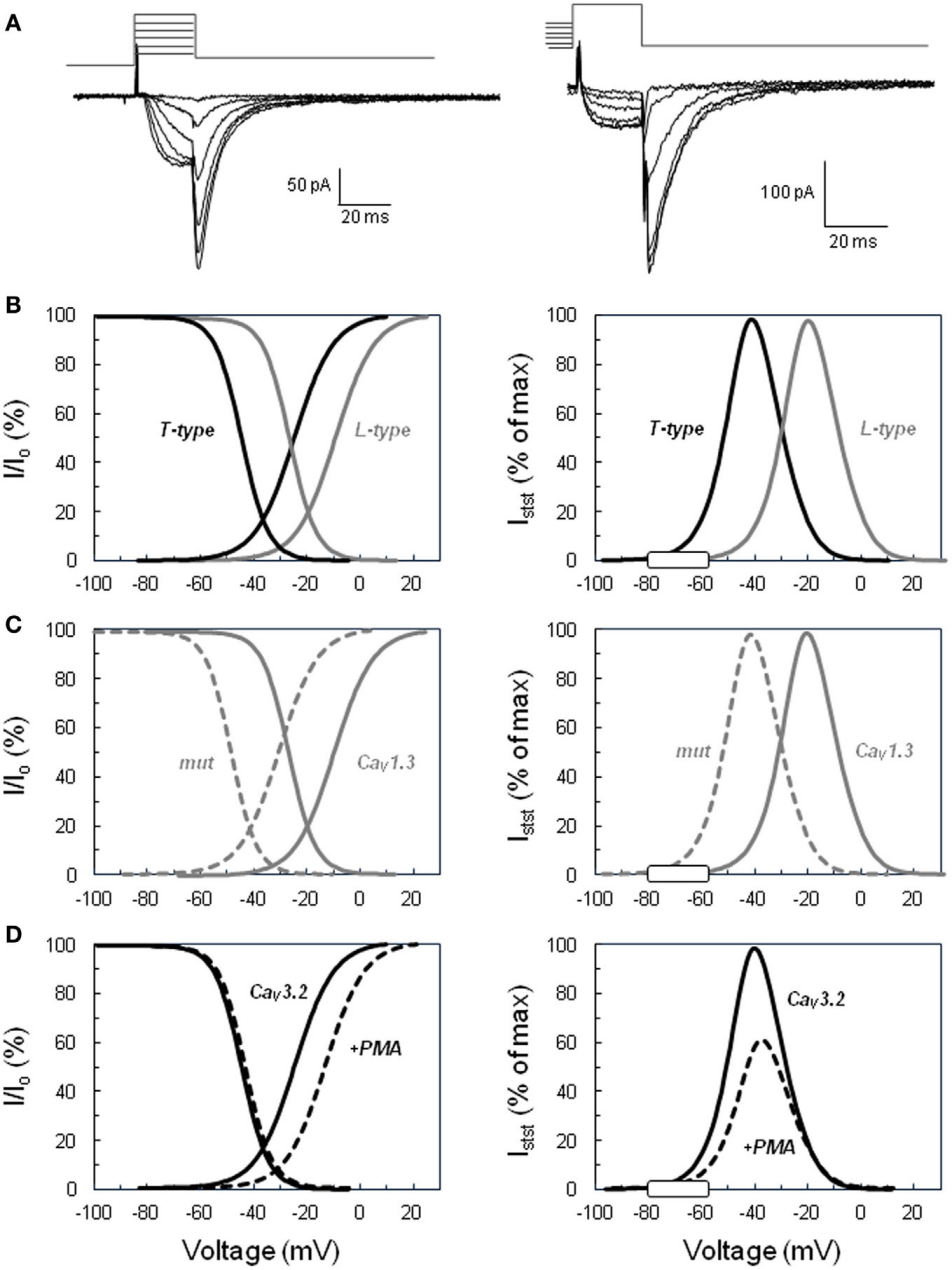
**Activation and inactivation of voltage-operated calcium channels and steady-state “window” currents**. **(A)** Examples of slowly deactivating (T-type) Ba^2+^ currents recorded in the whole cell configuration of the patch clamp technique. Left. Voltage protocol for determining the activation curve: tail currents were evoked by repolarizing the cell to −65 mV after a short period (20 ms) of depolarization at various voltages (−45 to +5 mV for this selection of traces) from a holding potential of −90 mV. Right. Voltage protocol for determining the steady-state inactivation curve: tail currents were elicited in the same cell at −65 mV, but after steady-state inactivation of the channels for 10 s at various holding potentials (here from −80 to −30 mV) and 20 ms activation at +20 mV. Current amplitude upon cell repolarization was then determined by fitting tail currents to an exponential function (the time constant was approximately 7 ms). **(B)** Comparison of low (T-type) versus high (L-type) threshold voltage-operated calcium channels. Left panel shows normalized activation and inactivation curves determined for T-type channels using the same type of protocol as shown in panel A. Tail current amplitudes were plotted as a function of the test voltage, fitted to Boltzmann’s equation, and normalized to the maximal current (*I*_o_). Curves for L-type channels were similarly defined from L-current amplitudes determined with a different voltage protocol, including the inactivation of T currents. Right panel displays the calculated normalized steady-state current (*I*_stst_) expected through T-type and L-type channels within their respective permissive window of voltage. The theoretical steady-state currents were obtained from the activation and inactivation curves according to Ohm’s law and expressed as a percentage of the maximal current. The white rectangle on the voltage axis indicates the range of membrane potentials reached in naive glomerulosa cells and in cells stimulated with physiological concentrations of angiotensin II or extracellular potassium. **(C)** Effect of the Ile770Met mutation described in the CACNA1D L-type calcium channel (13) on the channel activation, inactivation, and steady-state current. Curves have been determined as in **(B)** for the wild-type channel (continuous line, Ca_V_1.3) and for the mutant channel (dotted line, mut) and show the significant shift of the channel permissive window toward lower voltages. **(D)** Effect of PKC activation on CACNA1H T channel activation, inactivation, and steady-state current. Curves have been determined as in **(B)** for the naive channel (continuous line, Ca_V_3.2) and for the channel in glomerulosa cells treated with the PKC activator phorbol 12 myristate 13-acetate ester (dotted line, +PMA) and show the significant reduction of the amplitude of the maximal steady-state current with the slight shift of the permissive window toward higher voltages. The graphs of this figure have been constructed based on data available in Ref. (14, 13).

An important and discriminating property of T channels is their ability to activate upon small depolarization of the membrane, allowing a surge of calcium entry into excitable cells at the beginning of an action potential (when the electrochemical gradient is highly favorable for cation entry), as well as in only slightly depolarized non-excitable cells, like isolated adrenal cortex steroidogenic cells. Their rapid, voltage-dependent inactivation and their slow deactivation (Figure 1A) make their gating characteristics distinct from those of other channels. This feature not only allows isolating specifically the corresponding currents thanks to defined voltage patch clamp protocols but also is responsible for the specific role played by these channels in the modulation of cardiac and neuronal cell excitability (10, 11). In cells devoid of action potential, such as isolated adrenal glomerulosa cells in culture, a sustained calcium entry within a permissive window of voltage is possible thanks to a significant overlap between the activation and inactivation potential ranges of the channel (12).

Indeed, voltage-operated calcium channels are classically characterized by their activation curve, reflecting the probability of channel opening at various membrane potentials (and, therefore, the proportion of channels activating at each voltage), and their steady-state inactivation curve, showing the proportion of channels remaining available (not inactivated) at different voltages (Figure 1B). These curves are produced experimentally by measuring currents under specific protocols, normalizing them and mathematically fitting data to Boltzmann’s equation. Examination of these curves reveals, for each type of channel, the presence of a *permissive window of voltage*, in which activation and inactivation overlap. A *sustained* flux of calcium through the channels is theoretically possible in this window, where a significant proportion of channels are already activated but not yet completely inactivated. This window delimits the range of voltages over which a steady-state current can flow through the channels, and the relative amplitude of this current can be calculated as a function of voltage using Ohm’s law (15). It is important to realize at this point that, because only a small fraction of channels is open at any time in this mode (upon slight membrane depolarization), the current amplitude is tiny as compared to the maximal current observed within the same cell when all channels open together during a putative action potential or upon a strong depolarization. However, because the channel activation is *sustained* for minutes (due to lack of complete inactivation), calcium accumulated within the cell during this period is huge in comparison to the amount entering during a single action potential that leads the cell to voltages less favorable for calcium influx.

Low threshold T-type calcium channels activate (and inactivate) at lower voltages than high-threshold L-type calcium channels, and, as a consequence, also present their permissive window at lower voltages. In fact, any channel modification (through phosphorylation, binding of G protein or genetic mutation) affecting its activation and/or inactivation curves will result in a marked change of the properties of the steady state current. Indeed, not only the position of the window will be shifted under these conditions but also the maximal amplitude of the steady-state current, which depends on both the extent of the overlap of the activation and inactivation curves and on the electrochemical gradient for calcium entry.

The resting potential of glomerulosa cells from different species has been measured to be between −86 and −73 mV (16–19), values that are at the left edge of the T channel window (see Figure 1B, right panel), but farther from that of L-type channel. Values reported for fasciculata cells are between −76 and −66 mV (16, 20, 21), showing that fasciculata cells are slightly depolarized (by 8–10 mV) as compared to glomerulosa cells under resting conditions. Moreover, depolarization of the cells by *physiological concentrations* of agonists like AngII, ACTH, or potassium (see below) has been determined to be maximally 10–20 mV (18–20, 22), which is sufficient for increasing the steady-state current through T channels by several folds. Increasing extracellular potassium, progressively from low to supra-physiological concentrations, has been shown to increase aldosterone secretion in parallel to the size of the predicted T-channel steady-state calcium current (12).

Whether adrenal cortical cells are naturally excitable (i.e., able to generate action potentials) has been debated. Indeed, when cell to cell contacts are preserved, several authors observed low frequency action potentials in both resting and stimulated glomerulosa and fasciculata cells. *Isolated* glomerulosa cells have been conventionally considered as non-excitable because their membrane potential rests close to the equilibrium potential for potassium (16) and remains negative to −60 mV upon stimulation with AngII or physiological concentrations of potassium. In contrast, some mouse zona glomerulosa cells *within adrenal slices* spontaneously generate membrane potential oscillations of low periodicity (0.44 Hz), as shown under whole cell current clamp conditions (17). Similar properties of rat, rabbit, and cat glomerulosa, and fasciculata cells had been previously reported (16, 18, 23). Electrical activity has generally to be induced in silent cells by depolarization with secretagogues (AngII, ACTH, or potassium) or by injecting depolarizing current in the current clamp mode (16, 18, 20), which is coherent with thresholds for triggering the action potential observed above −64 mV (17). Spontaneous or evoked voltage oscillations appear insensitive to inhibition by tetrodotoxin or nifedipine, but completely abolished by low nickel concentrations, suggesting that they are supported by calcium currents carried by low threshold T-type calcium channels (Ca_V_3.2) (17, 20). Moreover, the frequency of the electrical oscillator is regulated positively by potassium and AngII (17), providing an additional way to these agonists for controlling steroid production.

Indeed, the presence of action potentials on top of the mechanism previously described as being responsible for the steady state calcium entry through T-type channels is expected to affect calcium signaling and therefore steroidogenesis. However, the exact role and influence of the spontaneous or evoked action potentials on steroid production (mostly measured experimentally from isolated cells, devoid of action potentials) have not been precisely and specifically quantified yet, probably given the strong relationship existing between this electrical activity and calcium entry. For example, it is not known whether a low but sustained entry of calcium is not more efficient for stimulating and maintaining steroid production than pulsatile peaks of high calcium with intermittent periods of channel inactivation.

### Calcium Channel Mutations in Primary Aldosteronism

Aldosterone secretion from adrenal glomerulosa cells is under the control of AngII and extracellular potassium (24, 25), and indirectly regulates blood pressure through stimulation of renal sodium reabsorption. A dysregulation of aldosterone production may lead to systemic hypertension and hypokalemia (26). Adrenal aldosterone-producing adenomas (APA) constitutively produce aldosterone (27) and are a common cause of severe hypertension. About 5–10% of patients referred to hypertension clinics have APA (28, 29), which are typically benign and well circumscribed, and their removal cures or ameliorates hypertension. The principal genetic cause of APA development [present in 40% of these tumors (30)] has been attributed to recurrent mutations in the KCNJ5 potassium channel, affecting its ion selectivity filter and therefore responsible for a sustained depolarization of the glomerulosa cell with the subsequent activation of low voltage-operated calcium channels (31). Other mutations associated to the presence of APA were identified by whole exome sequencing. Somatic mutations affected two members of the P-type ATPase gene family, ATP1A1 (the α1 subunit of the Na/K-ATPase) and ATP2B3 (the plasma membrane calcium ATPase), highlighting again the importance of maintaining a strongly negative resting potential and a low cytosolic calcium concentration in adrenal glomerulosa cells for maintaining a low basal aldosterone production (32).

More recently, one additional gene, coding for a calcium channel, was identified with somatic and germline mutations present in other APA tumors without KCNJ5 mutation (13). CACNA1D encodes the α1 (pore-forming) subunit of Ca_V_1.3, a high-threshold L-type calcium channel. CACNA1D mutations were identified in 9% of APA and represent the second most frequent cause of the disease (30). In contrast to patients with KCNJ5 mutations who are more frequently females, CACNA1D carriers are mostly males. Paradoxically, APA are generally composed of large cells with the morphological appearance of fasciculata cells (ZF-like cells) but still express markers of glomerulosa cells, including the enzymes required for aldosterone biosynthesis (33). While KCNJ5 mutations were associated with large ZF-like APA, CACNA1D mutations are present in small ZG-like APA, suggesting that the cellular composition influences the adenoma size (32, 34). Whether the resting potential of these cells, expected to be less negative in KCNJ5 mutant APAs (like in fasciculata cells) than in normal glomerulosa or in CACNA1D mutant cells, is involved in the determination of cell size and morphology remains to be demonstrated, which is probably a difficult task given the reported heterogeneity of the lesions.

**Figure 2:**
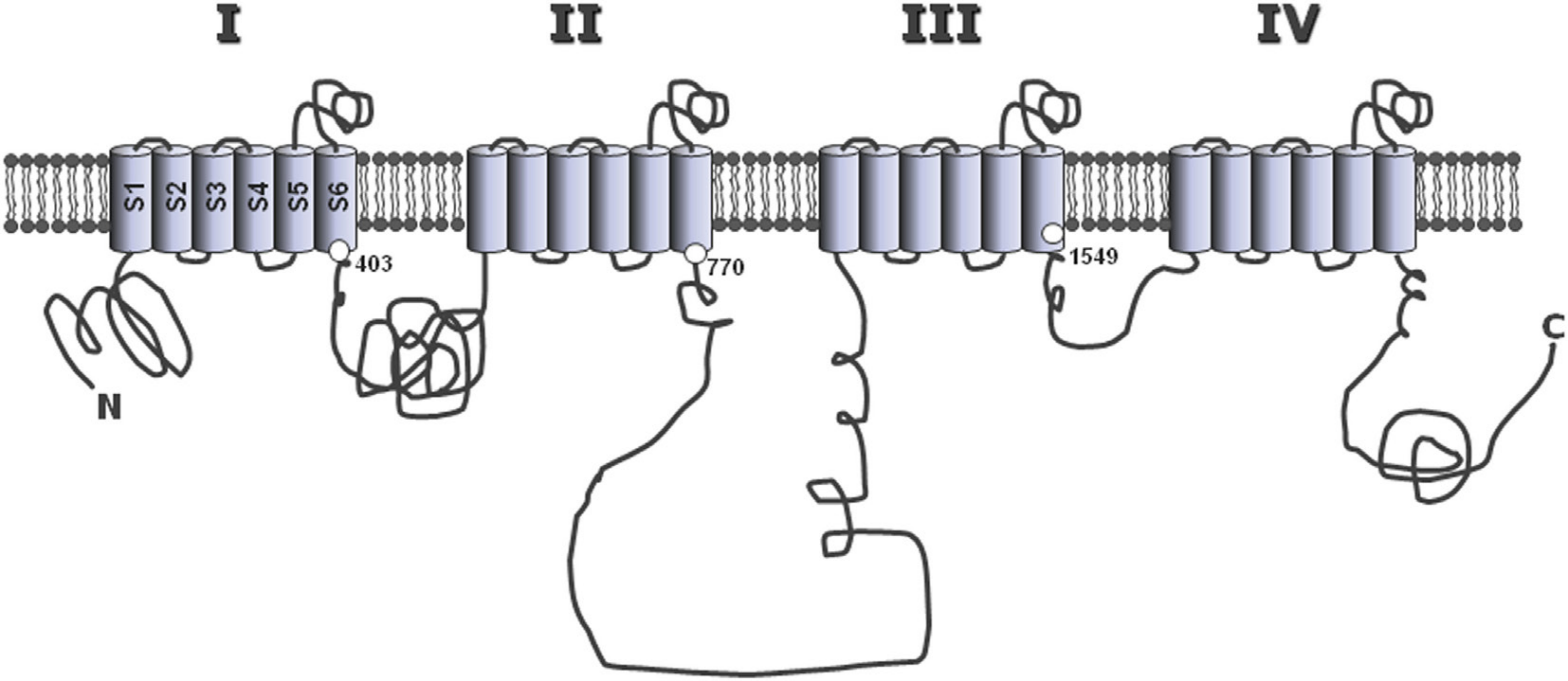
**Common structure of the alpha 1 subunit of voltage-operated calcium channels**. The main, pore-forming α_1_ subunits of the various voltage-operated calcium channels share a common general structure, with four homologous repeats (I–IV), each composed of six hydrophobic, putative membrane-spanning alpha helix domains (S1–S6). The three large loops connecting repeats together, as well as the N- and C-terminal extremities are located in the cytosol. The positions of Gly403 and Ile770 mutated in CACNA1D (13), and of Met1549 in CACNA1H (35), are indicated at the end of the S6 segments in repeats I, II, and III, respectively.

Mutations identified in CACNA1D and associated with APA were affecting two conserved amino acids of the L-type Ca_V_1.3 channel, Gly403 and Ile770, both located near the cytosolic end of S6 transmembrane segments in domains I and II (Figure 2), regions implicated in channel gating (13). To assess the effect of these mutations on channel function, wild type and mutant channels were expressed in HEK293T cells and their electrophysiological properties investigated with the patch clamp technique. Compared to wt channels, both mutants showed maximum current amplitude at less depolarized potentials, as well as a significant leftward shift (by 20 mV) of their activation and steady-state inactivation curves (Figure 1C). As a consequence, the permissive window of the channel is also shifted to more negative voltages, corresponding to membrane potentials reached upon physiological stimulations. In other words, these gain-of-function mutations of the channel, by shifting its threshold of activation, make the Ca_V_1.3 channel “T-like.”

Recently, using whole exome sequencing in subjects with unexplained primary aldosteronism (PA) diagnosed at a young age (<10 years), a new mutation has been identified that affect a T-type calcium channel (CACNA1H). Interestingly, the mutation (M1549V) is located within the S6 segment of domain III (Figure 2). Electrophysiology demonstrates that this variant causes a 10 times reduction of the rate of channel inactivation and a modest shift of the activation curve to more hyperpolarized potentials, two effects inferred to produce increased calcium influx and aldosterone hypersecretion (35).

Clearly, these observations highlight the importance of low threshold T-type calcium channels in the control of aldosterone biosynthesis, and they recently reactivated the general interest for a comprehensive understanding of the functional specificity of these channels in steroidogenesis. This review aims at recapitulating the arguments in favor of a privileged functional role for T channels in the control of steroid production, at comparing their involvement in aldosterone and cortisol secretion, and, finally, at discussing whether similar mutations on calcium channels, as observed in PA patients, are susceptible to occur in fasciculata cells and to be responsible of some forms of Cushing syndrome.

## Structure, Expression, and Properties of T-Type Calcium Channels in Adrenal Cortex

Although most studies of low-threshold T-type calcium current expression have focused on the analysis of neuronal and cardiac tissues, the presence of similar currents has been demonstrated throughout the body in both excitable and non-excitable cells, including adrenal glomerulosa (36–44) and fasciculata cells (45–48) from various species.

Three different genes code for the various T channels that are all composed of a single, large α_1_ subunit (49). According to the proposed nomenclature of voltage-gated calcium channels (50), α_1_G, α_1_H, and α_1_I T channel isoforms have been renamed Ca_V_3.1, 3.2, and 3.3, respectively (Table 1).

**Table 1:** **Nomenclature of T-type calcium channels**.

Channel name	Protein isoform	Gene name
Ca_V_3.1	α_1_G	CACNA1G
Ca_V_3.2	α_1_H	CACNA1H
Ca_V_3.3	α_1_I	CACNA1I

The expression in *Xenopus* oocytes or HEK293 cells of each of the three cloned α_1_ subunits coding for T channels generates typical low threshold transient currents with classical properties associated with T channels (51–54). Currents induced by Ca_V_3.1 and Ca_V_3.2 channels are nearly identical but can be easily distinguished by the higher sensitivity of Ca_V_3.2 to inhibition by nickel (55).

As for high threshold calcium channels and sodium channels (56, 57), the α_1_ subunits of T channels consist of four homologous repeats (I–IV), each being composed of six transmembrane segments (S1–S6), including a highly conserved pore loop (between S5 and S6) and a distinct voltage sensor (S4) (Figure 2). In contrast, more divergence between isoform sequences is found within the large intracellular loops, particularly that linking domains II and III, which also contains a site for alternative splicing in α_1_G (58). Interestingly, this variable region of the channel is responsible for the interaction of several calcium channels with specific cell proteins [such as the ryanodine receptor binding to the skeletal muscle L-type channel (59), or syntaxin binding to the neuronal N-type channel (60)], and could therefore participate to the establishment of specific functions for each channel isoform within the cell (see below).

The T channel family presents only 28% sequence identity with other voltage-gated calcium channels, while L (Ca_V_1.x) and non-L (Ca_V_2.x) high threshold channel families share more than 50% of their sequence. Replacement of two out of the four glutamate residues generally present in each P (pore-forming) region of the channel by aspartate seems to be responsible for the specific ion selectivity and permeation of T channels (61). Like for other calcium channels, molecular diversity among T channels is increased by alternative splicing of the mRNA of each of the three isotypes, and the properties of some of the 24 putative Ca_V_3.1 splice variants have been described by several groups (58, 62–64).

Physiological roles for T-type channels have been proposed in several tissues, including pacing of the heart (10), smooth muscle contraction (65), pain neurotransmission (5), fertilization (66), or adrenal steroid biosynthesis (38, 46). The tissue-selective expression of these channels and their isoforms has been determined at the molecular level by Northern blot analysis, *in situ* hybridization (ISH), RT-PCR, as well as by immunohistochemistry (IH) in human and other species. Ca_V_3.1 has been shown to be expressed predominantly in brain as well as in heart, while Ca_V_3.2 is mostly found in kidney and liver, but can be also observed in adrenal cortical cells.

The analysis of the expression pattern of the various T channel isoforms revealed that mRNA coding for each of the three α_1_ subunits is detectable in the adrenal gland, although in varying quantities. The molecular identity of the channels has been determined more specifically in the adrenal cortex, showing that the predominant subtype expressed in both glomerulosa and fasciculata cells from various species is Ca_V_3.2 (Table 2), a finding in agreement with the high sensitivity to nickel of the T currents recorded in these cells (67). In several studies, a stronger expression of the channel was reported to be present in glomerulosa cells as compare to fasciculata cells (35, 67, 68). Similarly, Ca_V_3.2 has been found to be the main isoform expressed in the human adrenocortical cell line H295R (69), but Ca_V_3.1 could be induced in the same cells by changing culture conditions (70).

**Table 2:** **Adrenal cortex expression of T-type calcium channel isotypes**.

Cell/tissue	Species	α_1_G/CaV3.1	α_1_H/CaV3.2
Adrenal zona glomerulosa	Bovine	–	Cloning/ISH (67)
Adrenal zona glomerulosa	Rat	–	ISH (67)
Adrenal zona fasciculata	Bovine		Northern blot (71)
Adrenal zona fasciculata	Human		Northern blot (72)
Adrenal cortex	Human		IH (35, 68)
H295R cells	Human	RT-PCR (70)	RT-PCR (69, 70)

Indeed, besides short-term modulation of T channels by hormones through the transduction mechanisms described below, a slower and more sustained regulation of channel activity results from the control of the expression of the α_1_ subunit. Such a regulation of T channel expression has been observed during the development of various organs, suggesting that these channels could play important roles at specific stages of the fetal life. The density of T channels has been shown to be relatively elevated in H295R cells, a cell line derived from a human adrenocarcinoma, as compared to corresponding normal tissue. This is reminiscent of the many pathological situations where re-expression of fetal genes has been observed during the evolution of oncologic diseases. Interestingly, aldosterone, which is produced by H295R cells, has been shown to significantly increase the T current amplitude, as well as the α_1_H transcript levels (69). Because T channels support steroidogenesis in various cells, including H295R cells, this autocrine action of aldosterone on channel expression represents a positive feedback loop that could help tumor cells to maintain differentiated functions like steroidogenesis.

Treatment of isolated rat (47) or bovine (71) fasciculata cells with ACTH for 3–6 days has been shown to induce the expression of CACNA1H mRNA and to markedly increase the amplitude of the T-type calcium currents, a response efficiently blocked by cycloheximide, an inhibitor of protein synthesis. ACTH treatment did not affect L-type current expression in these cells. Interestingly, the putative role of cAMP in this response to ACTH has been challenged by the use of distinct cellular messenger analogs and metabolites mimicking ACTH action without PKA activation (71, 73), and by the fact that ACTH-induced T current up-regulation was not affected by the PKA antagonist H89 (47).

Recently, the relative expression of four distinct calcium channels (Ca_V_1.2, 1.3, 2.2, and 3.2) has been analyzed at the mRNA and protein levels in human normal adrenal cortex, as well as in aldosterone (APA) and in cortisol-producing adenoma (CPA). While RT-PCR results showed that Ca_V_3.2 (CACNA1H) was the second most expressed channel in APA (after Ca_V_1.3), Ca_V_3.2 was clearly dominant in CPA (68), which is consistent with the prevalence of the T-current in fasciculata cell, as shown by electrophysiological recordings (45, 47). This also suggests a much stronger expression of T channels in CPA than in normal fasciculata cells. Moreover, in APA, only the T subunit Ca_V_3.2 was positively correlated among patients with individual peripheral blood aldosterone levels (68). In CPA cases, however, this correlation was not observed with cortisol levels.

The electrophysiological properties of endogenous T-type calcium channels have been extensively investigated with the patch clamp technique in rat (19, 47, 74), bovine (15, 45, 74–76), and human (43, 72) glomerulosa and fasciculata cells (Table 3), and compared to those of recombinant Ca_V_3.2 expressed in 293 cells (77). The threshold of current activation, the peak current voltage, and the half-maximal activation and steady-state inactivation (*V*_1/2_) were all within the range of voltages expected for low threshold T-type calcium channels. In other studies, using slow ramp depolarization, T current activation threshold in rat or bovine glomerulosa cells was determined to be at around −70 mV and that for L-type current at −55 mV (14, 78).

**Table 3:** **Electrophysiological properties of T channels in adrenocortical cells**.

	Glomerulosa	Fasciculata	Reference
Threshold of activation (mV)	−80 to −60	−70 to −50	(15, 19, 43, 45, 47, 74–76)
Peak current voltage (mV)	−40 to −30	−30 to −10	(19, 43, 45, 47, 72, 74–76)
Half-maximal activation *V*_1/2_ (mV)	−47 to −23	−50 to −17	(15, 19, 45, 47, 72, 76)
Half-maximal inactivation *V*_1/2_ (mV)	−74 to −50	−65 to −43	(15, 19, 45, 47, 72, 74, 76)

Interestingly, in spite of the fact that the same channel isoform as in glomerulosa cells (Ca_V_3.2) is apparently expressed in zona fasciculata (71, 72), what has been confirmed by the high sensitivity of the current to inhibition by nickel in these cells, all parameters appear slightly shifted toward more depolarized potentials in fasciculata cells. The reason for this difference is unknown and could be in part related to the selection of the reviewed literature.

The functional relevance of these observations, as previously mentioned, resides in the fact that, in the absence of action potentials and upon limited cell depolarization induced by low physiological concentrations of potassium or AngII, the membrane potential is expected to rapidly reach the region of the T channel permissive window, allowing calcium influx, but not that of L channels. As a consequence, T-type channels appear to be the main contributors to *sustained* calcium influx in response to AngII or physiological concentrations of potassium in glomerulosa cells, because the resting potential of these cells is highly negative, close to the potassium inversion potential (−80 mV), and the agonists do not depolarize the cell enough to reach the threshold of L-type channel activation (Figure 1B).

In contrast, it is probable that upon strong cell depolarization induced by supra-physiological concentrations of agonists, or under particular conditions, T channels discharge altogether, generating pulses of action potentials able to secondly activate high threshold L-type channels (75). However, the relative contribution to steroidogenesis of calcium entering the cell trough L-type channels in this mode appears quite limited, given the poor efficiency of dihydropyridines or other specific L-type channel calcium antagonists for inhibiting aldosterone or cortisol secretion, *in vivo* or *in vitro* (see below).

## Pharmacological Demonstration of T-Channel Function in Steroidogenesis

Adrenocortical T-type currents have been implicated early in the control of steroid biosynthesis (14, 38, 46, 79, 80), and this specific role for T channels has been extensively reviewed in the literature (12, 24, 81, 82). A direct demonstration of the crucial role played by these channels involved their inhibition by pharmacological agents. However, in spite of strong efforts to develop or isolate highly selective calcium antagonists during the last decades, only a few of the characterized pharmacological compounds appeared to preferentially affect T-type channels. Some of these drugs have been recently tested on heterologously expressed recombinant channels and sometimes display weak channel subtype selectivity. The reported potency (IC_50_) of these compounds on the various T channel isoforms is indicated in Table 4.

**Table 4:** **Pharmacology of T-type calcium channel isotypes**.

Agent (type/class)	Ca_V_3.1	Ca_V_3.2	Ca_V_3.3	Ca_V_1.x	Reference
IC_50_ (μM)					
Nickel (inorganic ion)	250–470	**5.4–13.0**	180–216	>200	(53, 55, 83–86)
Mibefradil (antihypertensive)	**0.12–1.20**	1.0–1.4	1.5	>12	(51, 53, 84, 87–90)
Phenytoin (anticonvulsant)	75–140	8–192	?	>360	(83, 91)
Propofol (anesthetic)	20	27	?		(91)
Pentobarbital (barbiturate)	310	345	?	>600	(91)
Kurtoxin (scorpion toxin)	**0.015**	0.061	?	>10	(92)
Pimozide (neuroleptic)	0.035	0.054	0.030		(93)
Flunarizine (neuroleptic)	**0.53 (*K*_D_)**	3.55 (*K*_D_)	0.84 (*K*_D_)		(93)
Anandamide (cannabinoid)	4.15	**0.33**	1.10		(94)
Amiloride (diuretics)		156–167			(53, 86)
Curcumin (condiment)		20			(95)
TTA-P2 (antinociceptive)		0.35		>3	(75)

*Bold indicates the channel isoform with the highest sensitivity to the most discriminant drugs*.

Inorganic polyvalent cations were among the first chemicals used to block T-type currents and their efficacy varies depending on the tissue examined, reflecting different channel isoform expression patterns (96). For example, nickel revealed to be useful for discriminating between Ca_V_3.2 (IC_50_ <15 μM) and Ca_V_3.1 (IC_50_ >250 μM). In contrast, the relative efficacy of zinc to inhibit T currents in various neuronal tissues (97, 98) suggests that this cation could have a higher affinity for Ca_V_3.1.

Mibefradil, a derivative of verapamil initially developed as a promising antihypertensive drug (99), and kurtoxin, a purified scorpion toxin (92), have been shown to preferentially inhibit T currents than high voltage-activated currents. Unfortunately, at the same concentrations, mibefradil also attenuates potassium currents (100), and kurtoxin interacts with sodium channels (92), therefore limiting their use for determining endogenous T channel function.

Dihydropyridines are widely used as antihypertensive drugs and are generally considered as specific L-type channel antagonists. However, some of these molecules also efficiently affect T channel activity. Indeed, while all these compounds inhibit L channels with similar potency (with IC_50_ around 0.1 μM), niguldipine and nicardipine are also efficient T channel antagonists, while nimodipine and nifedipine are much more useful for discriminating between L and T currents (101). As expected, dihydropyridine efficacy varies from one cell type to the other (96). Interestingly, while their binding affinity on L channels is clearly voltage-dependent, this dependence is much less pronounced in their interaction with T channels (102), suggesting that the selectivity of these drugs should also vary from one cell type to the other in part because of the difference in their resting potential.

Many other substances, such as succinimide derivatives, phenytoin, pimozide, flunarizine, zonisamide, barbiturates, some anesthetics, and benzodiazepines, employed in clinic essentially for treating neurological or psychiatric disorders, have been shown to also affect T channel activity in non-neuronal cells (96). However, it is noteworthy that none of the above mentioned pharmacological agents is absolutely selective for T-type channels and most of them also affect other types of ionic channels, particularly high threshold calcium channels, when employed at slightly higher concentrations.

The role of T channels in the control of steroidogenesis has been thoroughly investigated by comparing the effects of different classes of calcium antagonists. Aldosterone secretion by adrenal glomerulosa cells, either *in vivo*, or *in vitro* in response to AngII or potassium, is efficiently inhibited by several T channel blockers, like tetrandrine (103, 104), mibefradil (19, 105), zonisamide (14), peripheral-type benzodiazepines (106), nickel (104), efonidipine (107, 108), or nicardipine (109), but not by highly selective L channel antagonists (14, 79, 107, 108). The relative lack of effect *in vivo* of several dihydropyridine antihypertensive drugs on circulating aldosterone concentrations in human has been previously discussed (110). In a recent meta-analysis (111), T channel antagonists appeared more efficient than L channel blockers for reducing aldosterone secretion and improving renal function, but not for decreasing blood pressure.

An extensive pharmacological characterization of both T-type and L-type channels appears particularly relevant in cases of APA, not only for defining the treatment of hyperaldosteronism but also for diagnostic purposes. Indeed, if the cause of the disease is linked to a mutation on the potassium channel (KCNJ5), leading to sustained cell depolarization and calcium influx through T-type channels, most dihydropyridines will be expected to be quite inefficient for preventing aldosterone hypersecretion. In contrast, if a mutation on the L-type channel subunit α1D (CACNA1D) is causal (see above), and the mutant channel retains the same sensitivity to this class of calcium antagonists as the wild-type channel, the drugs will probably reduce aldosterone secretion. This hypothesis has been recently supported by the observation that a patient identified as affected by a *de novo* germline CACNA1D mutation responded particularly well to treatment with the dihydropyridine amlodipine, which normalized blood pressure and resolved ventricular hypertrophy in this patient (13).

Adrenocorticotropic hormone-induced cortisol secretion from bovine adrenal fasciculata cells has been similarly shown to be inhibited by low nickel concentrations (45). A parallel inhibition of T-type currents in these cells with an IC_50_ of 20 μM strongly suggests that the Ca_V_3.2 channel isoform is also mostly responsible for the steroidogenic response in fasciculata cells (45, 47). This was confirmed by the observation that mibefradil also inhibited both T-type calcium current and cortisol secretion induced by ACTH in bovine fasciculata cells with IC_50_’s of 1.0 and 3.5 μM, respectively (76). Curcumin directly modulates the activity of several types of ion channels, including Ca_V_3.2. In addition to a slight stimulatory effect on cortisol production from naive fasciculata cells, attributed to cell depolarization in response to potassium current inhibition, this compound has been shown to markedly reduce in parallel the large cortisol responses to both ACTH or AngII, and the Ca_V_3.2 current (95). Similar results were obtained recently with the organic compound TT-P2, a recently developed potent and quite selective antagonist of T-type calcium channels (75).

The relative lack of selectivity of most of these calcium antagonists has been partially circumvented by combining the information collected with several unrelated compounds and comparing their individual action on both T currents and steroid production in a comprehensive way (112). Indeed, the degree of aldosterone inhibition by a large series of calcium antagonists has been shown to be strongly correlated to the extent of T current inhibition, but not to that of L current inhibition (see below).

## Modulation of T-Channel Activity by Hormones

The unique signaling function of T channels suggests that their activity must be rapidly modulated by various hormones and extracellular modulators. While such a control of channel activity has been extensively described for high voltage-activated calcium channels (113, 114), much less information is available concerning T channels.

Nevertheless, rapid changes of T channel activity, positive or negative, have been reported in response to various agonists in several tissues, including adrenal cortical cells (Table 5).

**Table 5:** **T-type calcium channel modulation by hormones in adrenal cortex**.

Agonists	Tissue/cell type (species)	Effect	Cellular messenger	Reference
AngII	Adrenal glomerulosa (bovine)	+	Gi protein	(38, 42, 115)
AngII	Adrenal glomerulosa (bovine)	+	CaMKII	(116–118)
AngII	Adrenal glomerulosa (bovine)	–	PKC	(15, 119, 120)
ANP	Adrenal glomerulosa (bovine)	–	cGMP?	(40, 41, 121)
Dopamine	Adrenal glomerulosa (rat)	–	G prot (βγ)/PKA	(122, 123)
Serotonin	Adrenal glomerulosa (rat)	+	Gs/PKA	(124)
Endozepine	Adrenal (frog)	+	PKA	(125)
ACTH/AngII	Adrenal fasciculata (human)	+	n/a	(72)
ACTH/AngII	Adrenal fasciculata (bovine)	+	n/a	(46, 75)
ACTH/VIP	Adrenal fasciculata (rat)	+	n/a	(47)
Aldosterone	H295R (human)	+	Gene expression	(69)

For example, the effect of angiotensin II (AngII) on several different calcium channels has been thoroughly reviewed (126). Basically, the hormone can affect the activity of voltage-operated calcium channels either indirectly, by inducing cell depolarization, or directly, by modifying the channel intrinsic electrophysiological properties.

### Modulation of Channel Activity through Membrane Depolarization

A modulation by AngII of the glomerulosa cell membrane potential has been known for a long time (22). Indeed, application of AngII on isolated rat glomerulosa cells induces a biphasic response: a brief hyperpolarization phase followed by a sustained and reversible decrease of membrane conductance accompanied by a depolarization from the resting potential, estimated in these cells to be around −80 mV. This observation was confirmed by means of fluorescent probes for measuring membrane potential (127), or the patch clamp technique in the perforated patch configuration (19). As previously discussed, at low physiological concentrations, AngII did not evoke action potentials in naive glomerulosa cells, but only shifted the voltage by 10–20 mV. The hormone-induced cell depolarization observed in rat, bovine, and human glomerulosa cells was always due to the inhibition of potassium permeability (128), but some differences between species were seen in the characteristics of the potassium currents involved. Four distinct types of potassium channels were identified in rat and bovine glomerulosa cells (129), but AngII induced a substantial inhibition of only inward rectifier and delayed rectifier potassium channel activities in these species (130). This action was reversible and blocked by the AT1 receptor antagonist losartan (131, 132). In contrast, AngII has been shown to also inhibit a charybdotoxin-sensitive current in rat glomerulosa cells (133), suggesting that a large conductance calcium-activated (BK) potassium channel is also modulated by the hormone.

The modulation of potassium permeability by AngII has been also investigated in bovine glomerulosa cells by measuring the efflux of ^86^Rb or ^43^K (134–136). The authors found that after a transient activation that was sensitive to apamin, a blocker of calcium-sensitive (SK) potassium channels, the efflux was then inhibited in a concentration-dependent manner by AngII, and that changes in potassium conductance reflect changes in membrane potential. Thus, the transient hyperpolarization would be due to the activation of calcium-activated potassium channels responding to the transient elevation of cytosolic free calcium concentration ([Ca^2+^]_c_) occurring upon calcium release from intracellular stores, while other types of potassium channels, such as the delayed rectifier, are later inhibited in a sustained fashion and are responsible for the depolarization phase. The latter effect of AngII on the potassium permeability being mimicked by diacylglycerol, a role for PKC in this process has been suggested (137). The functional link between potassium conductance modulation and aldosterone secretion was also supported by the demonstration that AngII-induced steroidogenesis is markedly affected by potassium ionophores like valinomycin (138), or by various potassium channel antagonists (139) or agonists (140), and, more recently, by the demonstration of the role of the KCNJ5 channel in maintaining a very negative resting potential within these cells and therefore a low basal aldosterone production (31).

Similar results have been obtained with adrenal fasciculata cells. Indeed, in bovine cells, AngII induces a biphasic response corresponding to a transient hyperpolarization followed by a sustained depolarization (141). The hyperpolarization phase appears to be due to the activation of calcium-dependent potassium currents, but also to chloride currents, the resting potential of fasciculata cells being less negative than that of glomerulosa cells. The identity of the conductance responsible for the fasciculata cell depolarization upon AngII challenge is less clearly defined. A cholera toxin-sensitive potassium current has been proposed to maintain the resting potential of bovine fasciculata cells and to be reduced by AngII and ACTH (142). However other mechanisms have been also suggested, such as the activation of a non-selective cationic conductance, the capacitative calcium influx triggered by calcium release from the stores, or the stimulation of the electrogenic 3Na/1Ca exchanger by elevated [Ca^2+^]_c_.

It should be repeated here that the cell depolarization evoked by hormones like AngII or ACTH is sustained but very limited in amplitude. As previously discussed, the consequence therefore is a selective activation of low threshold T-type calcium channels, particularly in cells displaying a very negative resting potential. These particular conditions also explain the exquisite sensitivity of glomerulosa cells to small variations of extracellular potassium, within its physiological range of concentrations.

However, it is important to remember that isolating glomerulosa and fasciculata cells from the adrenal cortex could prevent the occurrence of spontaneous or evoked electrical activity. The presence of action potentials in these cells would allow bringing the membrane depolarization transiently up to voltage values corresponding to L-channel activation and T-channel inactivation and, therefore, the relative contribution of low and high voltage channels in the steroidogenic response to secretagogues could have been wrongly estimated in isolated cells devoid of AP. Nevertheless, because T channels are apparently involved in the triggering and the propagation of the putative action potentials required for L channel activation (17), their function remains crucial even in cells able to work in an “excitable mode.”

### Modulation of Channel Activity through Protein Modification

The modulation of calcium channels by G proteins has been recognized for a long time as one on the main mechanisms employed by hormones to control the influx of calcium into their target cells (3, 114, 143). In most cases, G proteins that interact with calcium channels are sensitive to pertussis toxin treatment. This toxin, which efficiently blocks the activation of both G_i_ and G_o_ proteins, therefore represents a valuable tool for determining the intracellular signaling pathway leading to channel modulation. Moreover, the interaction between the receptor, the G protein and the channel is generally considered to be restricted to a small area of the membrane because it can be observed in the excised patch configuration of the patch-clamp technique, in contrast to other mechanisms involving diffusible molecules acting on channels located at distance from the receptor. It is important to realize that, depending on the mechanism involved, a given receptor can influence calcium influx very locally or broadly within the cell, a distinction that can have physiological consequences depending on the structural and functional organization of the cell (see below).

The modulation of T-type channels by AngII in bovine adrenal glomerulosa cells has been the object of some controversy. Indeed, early observations suggest that, in these cells, AngII increases the amplitude of the slowly deactivating and rapidly inactivating calcium currents, linked to the T-type channel activity (38). Further studies revealed that AngII, in fact, induces a shift of the channel activation curve toward more negative potential values (making the channel more prone to opening upon limited cell depolarization), an effect requiring the presence of GTP in the patch pipette and mimicked by the addition of GTPγS (42). These results, as well as the fact that AngII action is prevented by pertussis toxin treatment or introduction of a monoclonal antibody generated against recombinant Gαi (115), are in favor of the involvement of a Gi protein in the activation of T channels. However, a direct interaction between the G protein and the channel is questioned by the observation that the hormone can increase the single channel opening probability in the cell attached configuration (42). Indeed, the latter observation strongly suggests that a rapidly diffusible second messenger is generated by AngII and responsible for the modulation of T channels. In fact the possibility that, in adrenal glomerulosa cells, AngII modulates T channel activity by more than one single mechanism has been raised by the following observation: although activation of CaMKII by the rise of intracellular calcium also shifts the activation curve of T channels to more negative potentials, as demonstrated with pharmacological inhibitors of the kinase (116), these inhibitors only minimally reduced AngII action (115).

In contrast, in another series of experiments, AngII has been shown to markedly *reduce* T channel activity in bovine glomerulosa cells through a PKC-mediated shift of the channel activation curve to more *positive* values (15). This inhibition of T channels was mimicked by PMA (Figure 1D) and correlated with the reduction of the potassium-induced aldosterone production observed in the presence of the same agent. The functional link between the T channel activity and aldosterone production evoked by extracellular potassium was further demonstrated by the observation that PMA did not affect either L-type channel activity (120) or steroidogenesis triggered with a calcium ionophores (14). This paradoxical inhibitory action of AngII is somehow balanced by the cell depolarization evoked by the hormone and can have some physiological relevance. Indeed, aldosterone production induced by extracellular potassium, which is highly dependent on the activity of T-type channels, is reduced in a PKC-dependent manner by AngII in rat glomerulosa cells, putatively to prevent overstimulation in the presence of both agonists (144).

The multiplicity of the mechanisms involved by a same hormone like AngII to modulate T currents, sometimes in opposite directions, could reflect the presence of various T channel isoforms within the cell under specific culture conditions. It would be therefore relevant to determine whether the expression of the various channel isoforms is changing during development or upon specific pathological states, in order to better predict the hormone action on calcium influx.

Several other physiological modulators of steroidogenesis and/or their intracellular messengers have been reported to affect T channel activity in adrenocortical cells (Table 5). Moreover, activation of ectopic receptors for serotonin or GIP, illegitimately expressed in human fasciculata cells, and causing Cushing syndrome through stimulation of cAMP in ACTH-independent macronodular adrenal hyperplasia, has been also reported to enhance T channel activity through a PKA-dependent mechanism (145).

Finally, it is noteworthy that channel modulation, being through phosphorylation or binding to G proteins, not only affects the electrophysiological properties of the channel but could also modify channel interaction with cellular proteins involved in the calcium signal transduction.

## Putative Mechanisms Conferring Functional Specificity to T-Channels in Steroidogenesis

As previously discussed, a close correlation has been observed in many instances between the production of aldosterone and the activity of T channels (but not L channels) upon modulation with potassium (12, 38), AngII (144), ANP (40), or several pharmacological agents affecting PKC (15, 144). Similarly, the steroidogenic response of glomerulosa cells to prolactin (103) or serotonin (124) has been shown to rely, at least partially, on the modulation of T channels, as well as the inhibition of aldosterone secretion by dopamine (122, 123, 146). The production of glucocorticoids by fasciculata cells was also dependent on T-type calcium channels when stimulated either by ACTH (46) or by an endozepine triakontetraneuropeptide (125).

Because a specific role in the modulation of steroid biosynthesis and secretion has been attributed to T-type channels in adrenal cortical cells, the challenge then consisted in understanding how their molecular and electrophysiological characteristics and how the structural organization of the cell confer a functional advantage to these channels.

In a few experiments, both T-type and L-type calcium currents have been simultaneously determined under various discriminating pharmacological conditions, and this concomitantly with measurement of cytosolic calcium fluctuations in the same single cell (14). Potassium-induced cytosolic calcium elevation and aldosterone production in response to the same agents were also determined in parallel. Steroidogenesis appeared systematically linked to T channel (but not to L channel) activity while cytosolic calcium fluctuations depended principally on L current amplitude (112). This finding suggests dissociation between the cytosolic calcium signaling evoked by extracellular potassium and the rate-limiting steps of aldosterone biosynthesis, known to occur within the mitochondria (147, 148). According to our working hypothesis, in order to exert their preferential stimulatory action on steroid biosynthesis, T channels have to fulfill at least two prerequisites: (a) maintain a sustained influx of calcium into the cell, and (b) direct a part of this calcium toward the mitochondria.

The first point is addressed by considering the particular electrophysiological properties of T channels. Indeed, how can transient (T-type) calcium currents, which inactivate within a fraction of a second in excitable cells, support activation of steroidogenesis in response to potassium for minutes or even hours? As previously discussed, upon slight depolarization of the cell, and in the absence of action potentials, a sustained entry of calcium within a permissive window of voltage is possible due to the significant overlap between the activation and inactivation potential ranges of the channel (12, 15, 40). This steady-state current is tiny and barely detectable with the conventional patch-clamp technique, but, because it is *sustained*, in the long run it allows a large amount of calcium to enter and accumulate within the cell, at least sufficiently for activating steroidogenesis. The resting potential of the rat and bovine glomerulosa cells has been estimated to be around −80 mV, a value close to the low voltage edge of this window. As a consequence, a small depolarization of the membrane, induced by a slight increase of extracellular potassium in the physiological range, may increase by several folds the steady-state influx of calcium through T channels (Figure 1B). While L-type channels also display a similar window of voltage, this is located at much more positive potentials, rarely reached under physiological conditions (19).

In addition to the establishment of a permissive window of voltage in which calcium can enter the cells near the resting potential, the specific function of T channels is probably also conferred by their localization within the cell membrane. Indeed, it should be noted here that, at supraphysiological cell depolarization, even in the absence of regenerative electrical activity, other types of calcium channels are simultaneously activated in the same cells, leading to an apparent redundancy of the calcium entry pathways. However, only a part of the functions attributed to T channels is actually shared by these other channels, which sometimes even exert opposite effects on steroidogenesis (79). This fact suggests that the cell is able to decipher the calcium signal specifically resulting from T channel activation. This is theoretically possible for two reasons: (1) the signal is confined due to the compartmentalization of calcium within the cell, and (2) there is a strict organization of the transduction mechanisms at the molecular level (149). In other words, the close environment of the channel (the proximity of specific calcium-binding protein and/or cellular organelles) dictates the function of calcium entering the cell at this precise location.

Mitochondria play a particular role in intracellular calcium homeostasis. Indeed, because of the very negative potential of their inner membrane (around −180 mV), and the presence of a specific calcium uniporter, they avidly take up calcium when its concentration rises in the cytosol. Mitochondrial calcium influx is rapidly balanced by an equivalent efflux of calcium out of the organelle, which is dependent on sodium exchange, but only up to a given cytosolic concentration called the “mitochondrial set-point” (147). Above this set-point, fluxes are no more balanced and calcium rapidly accumulates within the mitochondrial matrix. This allows the cytosolic calcium concentration to come back down to this critical set-point. This property of mitochondria makes them very efficient cytosolic buffers, able to attenuate large calcium transients within the cell. At the same time, the mitochondria are also a *target* for calcium, particularly in steroidogenic cells. Indeed, steroids are synthesized by successive oxidations of a common precursor, cholesterol. Some important enzymatic steps in this process require molecular oxygen and, for this reason, must occur within the mitochondria. The rate limiting step in steroid biosynthesis is indeed the intramitochondrial conversion of cholesterol to pregnenolone, which requires the transfer of the substrate from the cytosol across the double membrane into the organelle. This early step in steroidogenesis is controlled by cytosolic and mitochondrial calcium levels as shown by several independent experimental observations: (a) rising ambient calcium stimulates aldosterone production in permeabilized glomerulosa cells and this response is prevented by the addition of ruthenium red, a blocker of the mitochondrial calcium uniporter (148), (b) intracellular calcium stimulates both intramitochondrial cholesterol transfer (150) and the expression of the steroidogenic acute regulatory (StAR) protein (151), which is required for efficient cholesterol transport into the mitochondria, and (c) cytosolic calcium fluctuations evoked by AngII are relayed and amplified within the mitochondrial matrix (152).

Interestingly, when mitochondrial calcium fluctuations induced by AngII were recorded at the *single organelle* levels with a GFP-derived probe (153), all mitochondria of the same cell did not respond simultaneously to the hormone, but calcium hot spots appeared in different regions of the cell, at different time points, and in an apparent stochastic manner (112). This observation suggests that the mitochondrial calcium response does not result from calcium uptake upon diffusion of calcium across the entire cytosol, but more probably upon local calcium release from intracellular stores located in proximity of the organelles.

**Figure 3:**
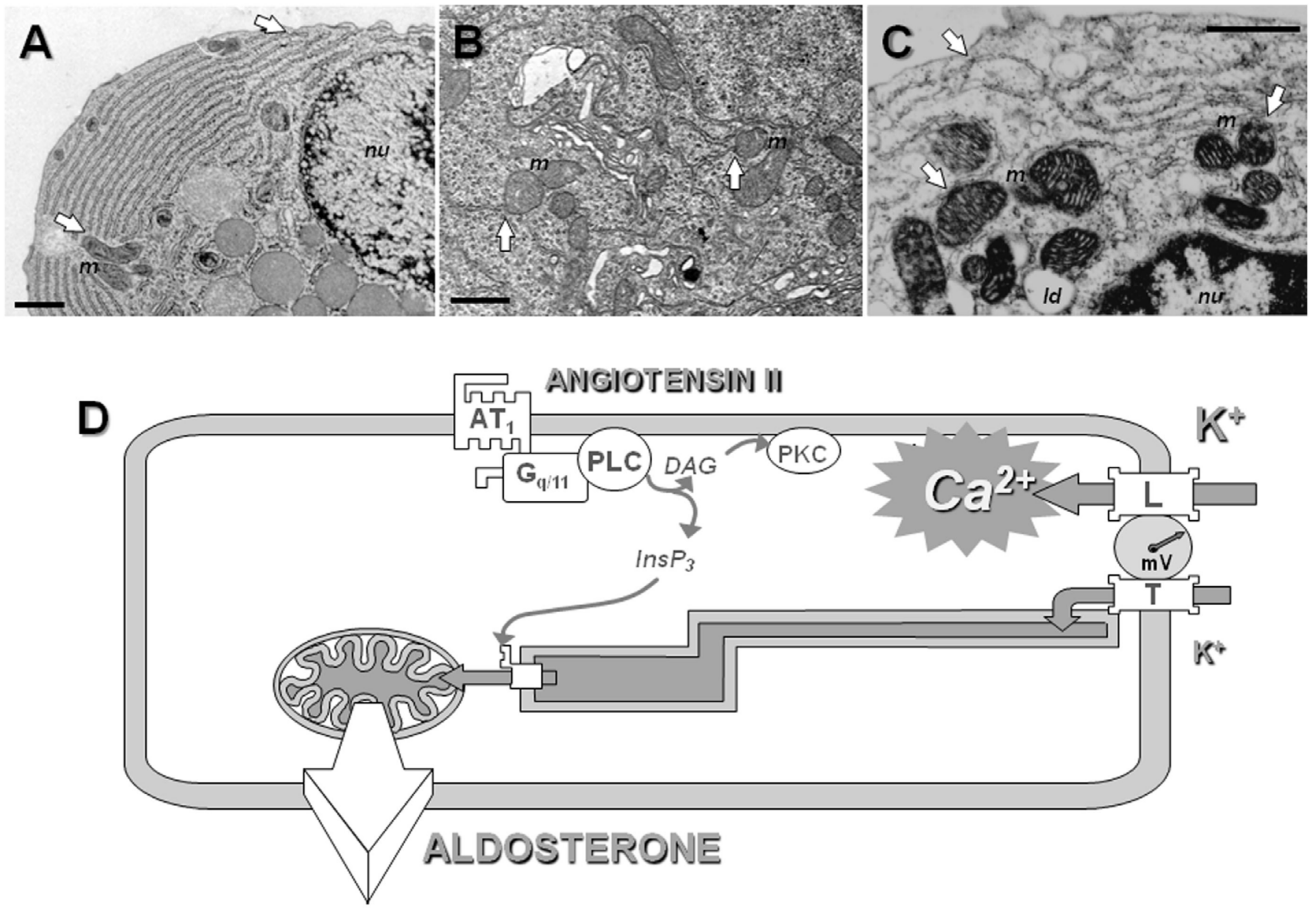
**The concept of intracellular calcium pipeline: a model for explaining the selective transport of calcium from the T channels into the mitochondria**. Electron microscopy reveals the presence of close apposition in many places of the endoplasmic reticulum (ER) with the plasma membrane or the mitochondria (white arrows), within various cell types, including rat parotid cells **(A)**, rat spinal cord neurons **(B)**, or bovine adrenal glomerulosa cells **(C)**. Scale = 1 μm; *m* indicates mitochondria, *nu* nucleus and *ld* lipid droplets. See Ref. (112) for additional information. **(D)**. A hypothetical model for the cellular transport of calcium into mitochondria. At the pipeline filling site, T-type calcium channels and, to a lesser extent, L-type channels are activated upon cell depolarization by potassium or angiotensin II. Several experimental data suggest that calcium entering the cell through T-type channels could be selectively pumped into the lumen of the ER, while calcium entering through L-type channels would be poured into the cytosol. At the pipeline delivery site, InsP3 receptors are maintained in proximity of the mitochondria within “quasi synaptic” structures. Calcium released upon activation of the InsP3 receptors, due to calcium overloading of the ER and/or to InsP3 production by AT_1_ receptor-activated PLC, is rapidly internalized into the very negatively charged matrix, through the mitochondrial inner membrane calcium uniporter. Intramitochondrial calcium elevation then stimulates limiting steps of aldosterone biosynthesis. AT_1_, angiotensin II receptor, type 1; G_q/11_, heterotrimeric G protein of the q/11 family; PLC, phospholipase C β; PKC, protein kinase C; InsP_3_, inositol 1,4,5-trisphosphate.

Because calcium stimulates early, rate limiting steps of steroidogenesis occurring within the mitochondria, and because calcium entering the cell through T channels is not detected within the cytosol of bovine adrenal glomerulosa cells (14), we have proposed that calcium is directly conveyed from the plasma membrane to the mitochondria *via* the lumen of the endoplasmic reticulum, which would act as a sort of “*intracellular calcium pipe-line*” (112, 149) (Figure 3).

This functional model is structurally supported by the distribution of the reticulum endoplasmic and the mitochondria within glomerulosa cells (and other cell types), with the presence of many “quasi synaptic” physical contacts observed between these organelles, as shown by electron microscopy, as well as by a close apposition of the reticulum with the plasma membrane in many points at the cell periphery (Figures 3A–C). This model therefore predicts specific interactions between T channels embedded within the plasma membrane and some components of the reticulum (Figure 3D). This proposal has been supported by the observation that disrupting physically the interaction between the reticulum and the cell membrane impairs the transduction of calcium signal elicited by potassium to the mitochondria, and therefore steroidogenesis in H295R cells (154). It is noteworthy that this organization of calcium influx, involving the local transfer of calcium entering the cell directly into the endoplasmic reticulum, is somehow reminiscent of the early “capacitative” model proposed by Putney (155), before revisiting it for taking into account results obtained with thapsigargin (156, 157), and it is quite intriguing that the α_1_H isoform of T channels, which is the most abundant in glomerulosa cells, has been proposed to mediate a capacitative calcium entry in non-excitable cancer cells (158).

The application of the pipeline model to the control of cortisol within fasciculata cells has not yet been specifically evaluated. The dichotomy between T-type and L-type channel functions is probably less obvious in these cells, where ACTH-induced steroid secretion is more efficiently inhibited by nifedipine and potentiated by Bay K8644, consistent with the involvement of L-type channels in the steroidogenic response (159, 160), in spite of the relatively low levels of expression of these channels reported in bovine fasciculata cells (45). However, species differences have been reported in term of dihydropyridine efficiency for reducing cortisol secretion (160). Clearly, additional investigations are still required for clarifying the functional relationship existing between calcium channels and limiting steps of cortisol biosynthesis, and therefore more specifically defining the role of each calcium channel.

## Conclusion

Two properties of T-type calcium channels appear to make them particularly suited for stimulating steroidogenesis: (1) their ability to activate and stay open upon slight depolarization of the cell, and (2) their capacity to convey calcium entering the cell directly to the mitochondria, where regulated steps of steroid biosynthesis occur.

The first property will be particularly favorable in cells displaying a very negative resting potential like glomerulosa cells, because small depolarization induced by physiological concentrations of agonists cannot reach the threshold for activation of L-type channels in these cells. The presence of these channels also renders the cells very sensitive to small changes of the extracellular potassium concentration, which participates, through the modulation of aldosterone secretion, to the maintenance of a normal kalemia. The fact that fasciculata cells have apparently a less negative resting potential probably could explain why steroidogenesis is globally less dependent on T-type channels in these cells and also partly involves L-type channel activity.

Because of calcium toxicity and polyvalence of calcium function, a strong confinement of this cation within the cell is necessary. The functional specificity of some channels is therefore also partly linked to their ability to transport calcium precisely to its site of action. In the case of steroidogenic cells, the mitochondrion is a main target for calcium. Because of the distance between the plasma membrane and these organelles in glomerulosa cells, the availability of intracellular calcium traffic through the lumen of the endoplasmic reticulum presents some advantages. Our working hypothesis is that T-type calcium channels are particularly efficient for stimulating aldosterone biosynthesis in glomerulosa cells because of their ability to load the endoplasmic reticulum with calcium. If the calcium pipeline model is true, one can predict that molecular structures must be maintained at both extremities and that T channels must interact physically with specific proteins. It is therefore conceivable that some mutations of the channel itself (or of the putative protein interacting with the channel) could prevent this interaction and therefore drastically reduce the ability of the channel to modulate steroidogenesis in spite of the fact that its electrophysiological properties are preserved. However, to our knowledge, such a loss of function mutation responsible for a hypoadrenocorticism has not been described.

The gain of function mutations on CACHA1D observed in APA cells could, theoretically, also affect cortisol secretion from fasciculata cells. Indeed, Ca_V_1.3 channels have been recently shown to be expressed in fasciculata cells and to participate to ACTH- and AngII-stimulated steroidogenic response (75). Similar mutations, shifting the channel permissive window to more negative voltages, would therefore be expected to increase cortisol secretion in an agonist-independent manner. Even if such mutations have not been formally demonstrated, the predicted high sensitivity of these tumors to inhibition by dihydropyridines would be worth of testing. Indeed, an important issue is whether the mutation status may influence the diagnosis, the treatment options, or the therapeutic outcome (32). Moreover, given the ubiquitous role played by calcium in cell biology and the prevalence of the diseases in which a dysfunction of calcium channels has been involved, a detailed understanding of the mechanisms conferring functional specificity to a given class of channels, like T-type channels, provides relevant information for developing strategies in order to get more efficient pharmacological tools in order to treat diseases resulting from calcium deregulation.

## Author Contributions

MR is responsible for the complete elaboration, writing, and correction of the present manuscript.

## Conflict of Interest Statement

The author has no commercial or financial relationship to disclose that could have influenced the redaction of the present review.

## Abbreviations

APA, aldosterone-producing adenoma; BK, broad conductance calcium-activated potassium channel; CPA, cortisol-producing adenoma; *I*_0_, maximal current; *I*_stst_, steady-state current; ISH, *in situ* hybridization; PA, primary aldosteronism; PMA, phorbol 12 myristate 13-acetate ester; SK, small conductance calcium-activated potassium channel; StAR, steroidogenic acute regulatory protein; ZF, zona fasciculata; ZG, zona glomerulosa.

## References

[1] Tsien RW Tsien RY Calcium channels, stores, and oscillations. Annu Rev Cell Biol (1990) 6:715–60. 10.1146/annurev.cb.06.110190.003435 2177344

[2] Trautwein W Hescheler J Regulation of cardiac L-type calcium current by phosphorylation and G proteins. Annu Rev Physiol (1990) 52:257–74. 10.1146/annurev.ph.52.030190.001353 2158764

[3] Schulz G Rosenthal W Hescheler J Trautwein W Role of G proteins in calcium channel modulation. Annu Rev Physiol (1990) 52:275–92. 10.1146/annurev.ph.52.030190.001423 1691905

[4] Rossier MF Function and differential expression of T-type calcium channels in various pathophysiological states. Recent Res Dev Biochem (2003) 4:13–29.

[5] Carbone E Lux HD A low voltage-activated, fully inactivating calcium channel in vertebrate sensory neurones. Nature (1984) 310:501–2. 10.1038/310501a0 6087159

[6] Nilius B Hess P Lansman JB Tsien RW A novel type of cardiac calcium channel in ventricular cells. Nature (1985) 316:443–6. 10.1038/316443a0 2410797

[7] Nowycky MC Fox AP Tsien RW Three types of neuronal calcium channels with different calcium agonist sensitivity. Nature (1985) 316:440–3. 10.1038/316440a0 2410796

[8] Perez-Reyes E Cribbs LL Daud A Lacerda AE Barclay J Williamson MP Molecular characterization of a neuronal low-voltage-activated T-type calcium channel. Nature (1998) 391:896–900. 10.1038/36110 9495342

[9] Perez-Reyes E. Molecular physiology of low-voltage-activated t-type calcium channels. Physiol Rev (2003) 83:117–61. 10.1152/physrev.00018.2002 12506128

[10] Hagiwara N Irisawa H Kameyama M. Contribution of two types of calcium currents to the pacemaker potentials of rabbit sino-atrial node cells. J Physiol (1988) 395:233–53. 10.1113/jphysiol.1988.sp016916 2457676 PMC1191991

[11] Huguenard JR. Low-threshold calcium currents in central nervous system neurons. Annu Rev Physiol (1996) 58:329–48. 10.1146/annurev.ph.58.030196.001553 8815798

[12] Rossier MF Burnay MM Capponi AM Distinct functions of T- and L-type calcium channels during activation of aldosterone production in adrenal glomerulosa cells. In: Clozel J-P Nargeot J Tsien RW, editors. Low Voltage-Activated T-Type Calcium Channels. Montpellier: Adis International (1998). p. 176–85.

[13] Scholl UI Goh G Stolting G de Oliveira RC Choi M Overton JD Somatic and germline CACNA1D calcium channel mutations in aldosterone-producing adenomas and primary aldosteronism. Nat Genet (2013) 45:1050–4. 10.1038/ng.2695 23913001 PMC3876926

[14] Rossier MF Burnay MM Vallotton MB Capponi AM Distinct functions of T-type and L-type calcium channels during activation of bovine adrenal glomerulosa cells. Endocrinology (1996) 137:4817–26. 10.1210/en.137.11.4817 8895352

[15] Rossier MF Aptel HBC Python CP Burnay MM Vallotton MB Capponi AM. Inhibition of low threshold calcium channels by angiotensin II in adrenal glomerulosa cells through activation of protein kinase C. J Biol Chem (1995) 270:15137–42. 10.1074/jbc.270.25.15137 7797497

[16] Quinn SJ Cornwall MC Williams GH. Electrical properties of isolated rat adrenal glomerulosa and fasciculata cells. Endocrinology (1987) 120:903–14. 10.1210/endo-120-3-903 3803318

[17] Hu C Rusin CG Tan Z Guagliardo NA Barrett PQ. Zona glomerulosa cells of the mouse adrenal cortex are intrinsic electrical oscillators. J Clin Invest (2012) 122:2046–53. 10.1172/JCI61996 22546854 PMC3966877

[18] Natke E Jr Kabela E Electrical response in cat adrenal cortex: possible relation to aldosterone secretion. Am J Physiol (1979) 237:E158–62. 464092 10.1152/ajpendo.1979.237.2.E158

[19] Lotshaw DP. Role of membrane depolarization and T-type Ca2+ channels in angiotensin II and K+ stimulated aldosterone secretion. Mol Cell Endocrinol (2001) 175:157–71. 10.1016/S0303-7207(01)00384-7 11325526

[20] Matthews EK Saffran M. Ionic dependence of adrenal steroidogenesis and ACTH-induced changes in the membrane potential of adrenocortical cells. J Physiol (1973) 234:43–64. 10.1113/jphysiol.1973.sp010333 4358269 PMC1350650

[21] Matthews EK. Membrane potential measurement in cells of the adrenal gland. J Physiol (1967) 189:139–48. 10.1113/jphysiol.1967.sp008159 16992241 PMC1396048

[22] Quinn SJ Cornwall MC Williams GH. Electrophysiological responses to angiotensin II of isolated rat adrenal glomerulosa cells. Endocrinology (1987) 120:1581–9. 10.1210/endo-120-3-903 3830061

[23] Matthews EK Saffran M Effect of ACTH on the electrical properties of adrenocortical cells. Nature (1968) 219:1369–70. 10.1038/2191369a0 4300402

[24] Capponi AM Rossier MF Regulation of aldosterone secretion. Curr Opin Endocrinol Diabetes (1996) 3:248–57. 10.1097/00060793-199606000-00010

[25] Capponi AM Rossier MF Angiotensin and aldosterone biosynthesis. In: Unger T Schölkens BA, editors. Angiotensin. Berlin: Springer (2004). p. 285–342.

[26] Vallotton MB Primary aldosteronism. Part I diagnosis of primary hyperaldosteronism. Clin Endocrinol (1996) 45:47–52. 8796138

[27] CONN JW Primary aldosteronism. J Lab Clin Med (1955) 45:661–4. 14368032

[28] Rossi GP Bernini G Caliumi C Desideri G Fabris B Ferri C A prospective study of the prevalence of primary aldosteronism in 1,125 hypertensive patients. J Am Coll Cardiol (2006) 48:2293–300. 10.1016/j.jacc.2006.07.059 17161262

[29] Hannemann A Wallaschofski H Prevalence of primary aldosteronism in patient’s cohorts and in population-based studies – a review of the current literature. Horm Metab Res (2012) 44:157–62. 10.1055/s-0031-1295438 22135219

[30] Fernandes-Rosa FL Williams TA Riester A Steichen O Beuschlein F Boulkroun S Genetic spectrum and clinical correlates of somatic mutations in aldosterone-producing adenoma. Hypertension (2014) 64:354–61. 10.1161/HYPERTENSIONAHA.114.03419 24866132

[31] Choi M Scholl UI Yue P Bjorklund P Zhao B Nelson-Williams C K+ channel mutations in adrenal aldosterone-producing adenomas and hereditary hypertension. Science (2011) 331:768–72. 10.1126/science.1198785 21311022 PMC3371087

[32] Zennaro MC Boulkroun S Fernandes-Rosa F. An update on novel mechanisms of primary aldosteronism. J Endocrinol (2015) 224:R63–77. 10.1530/JOE-14-0597 25424518

[33] Boulkroun S Samson-Couterie B Dzib JF Lefebvre H Louiset E Amar L Adrenal cortex remodeling and functional zona glomerulosa hyperplasia in primary aldosteronism. Hypertension (2010) 56:885–92. 10.1161/HYPERTENSIONAHA.110.158543 20937967

[34] Fischer E Beuschlein F. Novel genes in primary aldosteronism. Curr Opin Endocrinol Diabetes Obes (2014) 21:154–8. 10.1097/MED.0000000000000060 24739312

[35] Scholl UI Stolting G Nelson-Williams C Vichot AA Choi M Loring E Recurrent gain of function mutation in calcium channel CACNA1H causes early-onset hypertension with primary aldosteronism. Elife (2015) 4:e06315. 10.7554/eLife.06315 25907736 PMC4408447

[36] Matsunaga H Yamashita N Maruyama Y Kojima I Kurokawa K. Evidence for two distinct voltage-gated calcium channel currents in bovine adrenal glomerulosa cells. Biochem Biophys Res Commun (1987) 149:1049–54. 10.1016/0006-291X(87)90514-6 2447886

[37] Matsunaga H Maruyama Y Kojima I Hoshi T Transient calcium channel current characterized by a low threshold voltage in zona glomerulosa cells of rat adrenal cortex. Pflugers Arch (1987) 408:351–5. 10.1007/BF00581128 2438640

[38] Cohen CJ McCarthy RT Barrett PQ Rasmussen H Calcium channels in adrenal glomerulosa cells: potassium and angiotensin II increase T-type calcium current. Proc Natl Acad Sci USA (1988) 85:2412–6. 10.1073/pnas.85.7.2412 2451250 PMC280003

[39] Durroux T Gallo-Payet N Payet MD. Three components of the calcium current in cultured glomerulosa cells from rat adrenal gland. J Physiol (1988) 404:713–29. 10.1113/jphysiol.1988.sp017315 2473202 PMC1190851

[40] Barrett PQ Isales CM Bollag WB McCarthy RT Calcium channels and aldosterone secretion: modulation by potassium and atrial natriuretic peptide. Am J Physiol (1991) 261:F706–19. 1718167 10.1152/ajprenal.1991.261.4.F706

[41] Barrett PQ Isales CM Bollag WB McCarthy RT. Modulation of Ca2+ channels by atrial natriuretic peptide in the bovine adrenal glomerulosa cell. Can J Physiol Pharmacol (1991) 69:1553–60. 10.1139/y91-231 1663816

[42] McCarthy RT Isales CM Rasmussen H T-type calcium channels in adrenal glomerulosa cells: GTP-dependent modulation by angiotensin II. Proc Nat Acad Sci U S A (1993) 90:3260–4. 10.1073/pnas.90.8.3260 PMC46279 8386369

[43] Payet MD Durroux T Bilodeau L Guillon G Gallo-Payet N Characterization of potassium and calcium ionic currents in glomerulosa cells from human adrenal glands. Endocrinology (1994) 134:2589–98. 10.1210/endo.134.6.7515004 7515004

[44] Varnai P Osipenko ON Vizi ES Spät A. Activation of calcium current in voltage-clamped rat glomerulosa cells by potassium ions. J Physiol (1995) 483:67–78. 10.1113/jphysiol.1995.sp020568 7776242 PMC1157872

[45] Mlinar B Biagi BA Enyeart JJ Voltage-gated transient currents in bovine adrenal fasciculata cells. I. T-type Ca2+ current. J Gen Physiol (1993) 102:217–37. 8228909 10.1085/jgp.102.2.217 PMC2229150

[46] Enyeart JJ Mlinar B Enyeart JA T-type Ca2+ channels are required for adrenocorticotropin-stimulated cortisol production by bovine adrenal zona fasciculata cells. Mol Endocrinol (1993) 7:1031–40. 10.1210/me.7.8.1031 8232302

[47] Barbara JG Takeda K. Voltage-dependent currents and modulation of calcium channel expression in zona fasciculata cells from rat adrenal gland. J Physiol (1995) 488(Pt 3):609–22. 10.1113/jphysiol.1995.sp020994 8576852 PMC1156728

[48] Guyot A Dupre-Aucouturier S Ojeda C Rougier O Bilbaut A. Two types of pharmacologically distinct Ca(2+) currents with voltage-dependent similarities in zona fasciculata cells isolated from bovine adrenal gland. J Membr Biol (2000) 173:149–63. 10.1007/s002320001016 10630930

[49] Lambert RC Maulet Y Mouton J Beattie R Volsen S De Waard M T-type Ca2+ current properties are not modified by Ca2+ channel beta subunit depletion in nodosus ganglion neurons. J Neurosci (1997) 17:6621–8. 9254674 10.1523/JNEUROSCI.17-17-06621.1997 PMC6573151

[50] Ertel EA Campbell KP Harpold MM Hofmann F Mori Y Perez-Reyes E Nomenclature of voltage-gated calcium channels. Neuron (2000) 25:533–5. 10.1016/S0896-6273(00)81057-0 10774722

[51] Klugbauer N Marais E Lacinova L Hofmann F A T-type calcium channel from mouse brain. Pflugers Arch (1999) 437:710–5. 10.1007/s004240050836 10087148

[52] Klockner U Lee JH Cribbs LL Daud A Hescheler J Pereverzev A Comparison of the Ca2 + currents induced by expression of three cloned alpha1 subunits, alpha1G, alpha1H and alpha1I, of low-voltage-activated T-type Ca2 + channels. Eur J Neurosci (1999) 11:4171–8. 10.1046/j.1460-9568.1999.00849.x 10594642

[53] Williams ME Washburn MS Hans M Urrutia A Brust PF Prodanovich P Structure and functional characterization of a novel human low-voltage activated calcium channel. J Neurochem (1999) 72:791–9. 10.1046/j.1471-4159.1999.0720791.x 9930755

[54] Lee JH Daud A Cribbs LL Lacerda AE Pereverzev A Klockner U Cloning and expression of a novel member of the low voltage-activated T-type calcium channel family. J Neurosci (1999) 19:1912–21. 10066244 10.1523/JNEUROSCI.19-06-01912.1999 PMC6782566

[55] Lee JH Gomora JC Cribbs LL Perez-Reyes E. Nickel block of three cloned T-type calcium channels: low concentrations selectively block alpha1H. Biophys J (1999) 77:3034–42. 10.1016/S0006-3495(99)77134-1 10585925 PMC1300574

[56] Hofmann F Biel M Flockerzi V Molecular basis for calcium channel diversity. Annu Rev Neurosci (1994) 17:399–418. 10.1146/annurev.ne.17.030194.002151 8210181

[57] Catterall WA. Structure and function of voltage-gated ion channels. Trends Neurosci (1993) 16:500–6. 10.1016/0166-2236(93)90193-P 7509519

[58] Cribbs LL Gomora JC Daud AN Lee JH Perez-Reyes E. Molecular cloning and functional expression of Ca(v)3.1c, a T-type calcium channel from human brain. FEBS Lett (2000) 466:54–8. 10.1016/S0014-5793(99)01756-1 10648811

[59] Tanabe T Beam KG Adams BA Niidome T Numa S. Regions of the skeletal muscle dihydropyridine receptor critical for excitation-contraction coupling. Nature (1990) 346:567–9. 10.1038/346567a0 2165570

[60] Sheng Z-H Rettig J Cook T Catterall WA. Calcium-dependent interaction of N-type calcium channels with the synaptic core complex. Nature (1996) 379:451–4. 10.1038/379451a0 8559250

[61] Talavera K Staes M Janssens A Klugbauer N Droogmans G Hofmann F Aspartate residues of the Glu-Glu-Asp-Asp (EEDD) pore locus control selectivity and permeation of the T-type Ca(2+) channel alpha(1G). J Biol Chem (2001) 276:45628–35. 10.1074/jbc.M103047200 11526105

[62] Mittman S Guo J Agnew WS. Structure and alternative splicing of the gene encoding alpha1G, a human brain T calcium channel alpha1 subunit. Neurosci Lett (1999) 274:143–6. 10.1016/S0304-3940(99)00716-8 10548410

[63] Monteil A Chemin J Bourinet E Mennessier G Lory P Nargeot J. Molecular and functional properties of the human alpha(1G) subunit that forms T-type calcium channels. J Biol Chem (2000) 275:6090–100. 10.1074/jbc.C000090200 10692398

[64] Jagannathan S Punt EL Gu Y Arnoult C Sakkas D Barratt CLR Identification and localization of T-type voltage-operated calcium channel subunits in human male germ cells. Expression of multiple isoforms. J Biol Chem (2002) 277:8449–56. 10.1074/jbc.M105345200 11751928

[65] Akaike N Kanaide H Kuga T Nakamura M Sadoshima J Tomoike H. Low-voltage-activated calcium current in rat aorta smooth muscle cells in primary culture. J Physiol (1989) 416:141–60. 10.1113/jphysiol.1989.sp017754 2558173 PMC1189208

[66] Arnoult C Cardullo RA Lemos JR Florman HM. Activation of mouse sperm T-type Ca2+ channels by adhesion to the egg zona pellucida. Proc Natl Acad Sci U S A (1996) 93:13004–9. 10.1073/pnas.93.23.13004 8917534 PMC24036

[67] Schrier AD Wang H Talley EM Perez-Reyes E Barrett PQ The alpha1 H T-type calcium channel is the predominant subtype expressed in bovine and rat zona glomerulosa. Am J Physiol (2001) 280:C265–72. 10.1152/ajpcell.2001.280.2.C265 11208520

[68] Felizola SJ Maekawa T Nakamura Y Satoh F Ono Y Kikuchi K Voltage-gated calcium channels in the human adrenal and primary aldosteronism. J Steroid Biochem Mol Biol (2014) 144(Pt B):410–6. 25151951 10.1016/j.jsbmb.2014.08.012

[69] Lesouhaitier O Chiappe A Rossier MF. Aldosterone increases T-type calcium currents in human adrenocarcinoma (H295R) cells by inducing channel expression. Endocrinology (2001) 142:4320–30. 10.1210/endo.142.10.8435 11564691

[70] Rossier MF Lesouhaitier O Perrier E Bockhorn L Chiappe A Lalevee N. Aldosterone regulation of T-type calcium channels. J Steroid Biochem Mol Biol (2003) 85:383–8. 10.1016/S0960-0760(03)00201-2 12943726

[71] Liu H Enyeart JA Enyeart JJ ACTH induces Cav3.2 current and mRNA by cAMP-dependent and cAMP-independent mechanisms. J Biol Chem (2010) 285:20040–50. 10.1074/jbc.M110.104190 20424171 PMC2888416

[72] Enyeart JJ Enyeart JA. Ca2+ and K+ channels of normal human adrenal zona fasciculata cells: properties and modulation by ACTH and AngII. J Gen Physiol (2013) 142:137–55. 10.1085/jgp.201310964 23858003 PMC3727308

[73] Enyeart JA Liu H Enyeart JJ 8-Phenylthio-adenines stimulate the expression of steroid hydroxylases, Ca_v_3.2 Ca^2+^ channels, and cortisol synthesis by a cAMP-independent mechanism. Am J Physiol Endocrinol Metab (2011) 301:E941–54. 10.1152/ajpendo.00282.2011 21810929 PMC3213996

[74] Quinn SJ Brauneis U Tillotson DL Cornwall MC Williams GH. Calcium channels and control of cytosolic calcium in rat and bovine zona glomerulosa cells. Am J Physiol (1992) 262:C598–606. 1312778 10.1152/ajpcell.1992.262.3.C598

[75] Enyeart JJ Enyeart JA. Adrenal fasciculata cells express T-type and rapidly and slowly activating L-type Ca2+ channels that regulate cortisol secretion. Am J Physiol Cell Physiol (2015) 308:C899–918. 10.1152/ajpcell.00002.2015 25788571 PMC4451351

[76] Gomora JC Xu L Enyeart JA Enyeart JJ. Effect of mibefradil on voltage-dependent gating and kinetics of T-type Ca(2+) channels in cortisol-secreting cells. J Pharmacol Exp Ther (2000) 292:96–103. 10604935

[77] Wolfe JT Wang H Perez-Reyes E Barrett PQ Stimulation of recombinant Ca(v)3.2, T-type, Ca(2+) channel currents by CaMKIIgamma(C). J Physiol (2002) 538:343–55. 11790804 10.1113/jphysiol.2001.012839 PMC2290082

[78] Varnai P Petheo GL Makara JK Spat A. Electrophysiological study on the high K+ sensitivity of rat glomerulosa cells. Pflugers Arch (1998) 435:429–31. 10.1007/s004240050534 9426301

[79] Barrett PQ Ertel EA Smith MM Nee JJ Cohen CJ. Voltage-gated calcium currents have two opposing effects on the secretion of aldosterone. Am J Physiol (1995) 268:C985–92. 7733247 10.1152/ajpcell.1995.268.4.C985

[80] Chen XL Bayliss DA Fern RJ Barrett PQ A role for T-type calcium channels in synergistic control of aldosterone production by angiotensin II and potassium. Am J Physiol (1999) 276:F674–83. 10330049 10.1152/ajprenal.1999.276.5.F674

[81] Spat A Rohacs T Horvath A Szabadkai G Enyedi P. The role of voltage-dependent calcium channels in angiotensin-stimulated glomerulosa cells. Endocr Res (1996) 22:569–76. 10.1080/07435809609043748 8969913

[82] Spat A Hunyady L. Control of aldosterone secretion: a model for convergence in cellular signaling pathways. Physiol Rev (2004) 84:489–539. 10.1152/physrev.00030.2003 15044681

[83] Lacinova L Klugbauer N Hofmann F. Regulation of the calcium channel alpha(1G) subunit by divalent cations and organic blockers. Neuropharmacol (2000) 39:1254–66. 10.1016/S0028-3908(99)00202-6 10760367

[84] Perchenet L Benardeau A Ertel EA. Pharmacological properties of Ca(V)3.2, a low voltage-activated Ca2+ channel cloned from human heart. Naunyn Schmiedebergs Arch Pharmacol (2000) 361:590–9. 10.1007/s002100000238 10882033

[85] Monteil A Chemin J Leuranguer V Altier C Mennessier G Bourinet E Specific properties of T-type calcium channels generated by the human alpha 1I subunit. J Biol Chem (2000) 275:16530–5. 10.1074/jbc.C000090200 10749850

[86] Berthier C Monteil A Lory P Strube C. Alpha(1H) mRNA in single skeletal muscle fibres accounts for T-type calcium current transient expression during fetal development in mice. J Physiol (2002) 539:681–91. 10.1113/jphysiol.2001.013246 11897840 PMC2290181

[87] Cribbs LL Lee JH Yang J Satin J Zhang Y Daud A Cloning and characterization of alpha1H from human heart, a member of the T-type calcium channel gene family. Circ Res (1998) 83:103–9. 10.1161/01.RES.83.1.103 9670923

[88] Jimenez C Bourinet E Leuranguer V Richard S Snutch TP Nargeot J. Determinants of voltage-dependent inactivation affect Mibefradil block of calcium channels. Neuropharmacol (2000) 39:1–10. 10.1016/S0028-3908(99)00153-7 10665814

[89] Mehrke G Zong XG Flockerzi V Hofmann F The calcium channel blocker Ro 40-5967 blocks differently T-type and L-type calcium channels. J Pharmacol Exp Ther (1994) 271:1483–8. 7996461

[90] Martin RL Lee JH Cribbs LL Perez-Reyes E Hanck DA. Mibefradil block of cloned T-type calcium channels. J Pharmacol Exp Ther (2000) 295:302–8. 10991994

[91] Todorovic SM Perez-Reyes E Lingle CJ. Anticonvulsants but not general anesthetics have differential blocking effects on different T-type current variants. Mol Pharmacol (2000) 58:98–108. 10860931 10.1124/mol.58.1.98

[92] Chuang RS Jaffe H Cribbs L Perez-Reyes E Swartz KJ. Inhibition of T-type voltage-gated calcium channels by a new scorpion toxin. Nat Neurosci (1998) 1:668–74. 10.1038/3669 10196582

[93] Santi CM Cayabyab FS Sutton KG McRory JE Mezeyova J Hamming KS Differential inhibition of T-type calcium channels by neuroleptics. J Neurosci (2002) 22:396–403. 11784784 10.1523/JNEUROSCI.22-02-00396.2002 PMC6758663

[94] Chemin J Monteil A Perez-Reyes E Nargeot J Lory P. Direct inhibition of T-type calcium channels by the endogenous cannabinoid anandamide. EMBO J (2001) 20:7033–40. 10.1093/emboj/20.24.7033 11742980 PMC125779

[95] Enyeart JA Liu H Enyeart JJ Curcumin inhibits ACTH- and angiotensin II-stimulated cortisol secretion and Cav3.2 current. J Nat Prod (2009) 72:1533–7. 10.1021/np900227x 19653644 PMC2853174

[96] Yunker AM Modulation and pharmacology of low voltage-activated (“T-Type”) calcium channels. J Bioenerg Biomembr (2003) 35:577–98. 10.1023/B:JOBB.0000008024.77488.48 15000521

[97] Kaneda M Akaike N. The low-threshold Ca current in isolated amygdaloid neurons in the rat. Brain Res (1989) 497:187–90. 10.1016/0006-8993(89)90987-6 2790453

[98] Takahashi K Akaike N. Calcium antagonist effects on low-threshold (T-type) calcium current in rat isolated hippocampal CA1 pyramidal neurons. J Pharmacol Exp Ther (1991) 256:169–75. 1846413

[99] Ertel SI Clozel J-P Mibefradil (Ro 40-5967): the first selective T-type calcium channel blocker. Exp Opin Investig Drugs (1997) 6:569–82. 10.1517/13543784.6.5.569 15989621

[100] Liu JH Bijlenga P Occhiodoro T Fischer-Lougheed J Bader CR Bernheim L. Mibefradil (Ro 40-5967) inhibits several Ca2+ and K+ currents in human fusion-competent myoblasts. Br J Pharmacol (1999) 126:245–50. 10.1038/sj.bjp.0702321 10051142 PMC1565812

[101] Stengel W Jainz M Andreas K. Different potencies of dihydropyridine derivatives in blocking T-type but not L-type Ca2+ channels in neuroblastoma-glioma hybrid cells. Eur J Pharmacol (1998) 342:339–45. 10.1016/S0014-2999(97)01495-7 9548406

[102] Furukawa T Nukada T Miura R Ooga K Honda M Watanabe S Differential blocking action of dihydropyridine Ca2+ antagonists on a T-type Ca2+ channel (alpha1G) expressed in *Xenopus* oocytes. J Cardiovasc Pharmacol (2005) 45:241–6. 10.1097/01.fjc.0000154374.88283.15 15725949

[103] Kau MM Lo MJ Tsai SC Chen JJ Pu HF Chien EJ Effects of prolactin on aldosterone secretion in rat zona glomerulosa cells. J Cell Biochem (1999) 72:286–93. 10.1002/(SICI)1097-4644(19990201)72:2<286::AID-JCB13>3.0.CO;2-4 10022511

[104] Rossier MF Python CP Capponi AM Schlegel W Kwan CY Vallotton MB. Blocking T-type calcium channels with tetrandrine inhibits steroidogenesis in bovine adrenal glomerulosa cells. Endocrinology (1993) 132:1035–43. 10.1210/en.132.3.1035 8382595

[105] Rossier MF Ertel EA Vallotton MB Capponi AM. Inhibitory action of mibefradil on calcium signaling and aldosterone synthesis in bovine adrenal glomerulosa cells. J Pharmacol Exp Ther (1998) 287:824–31. 9864260

[106] Python CP Rossier MF Vallotton MB Capponi AM. Peripheral-type benzodiazepines inhibit calcium channels and aldosterone production in adrenal glomerulosa cells. Endocrinology (1993) 132:1489–96. 10.1210/endo.132.4.8384990 8384990

[107] Okayama S Imagawa K Naya N Iwama H Somekawa S Kawata H Blocking T-type Ca2+ channels with efonidipine decreased plasma aldosterone concentration in healthy volunteers. Hypertens Res (2006) 29:493–7. 10.1291/hypres.29.493 17044661

[108] Tanaka T Tsutamoto T Sakai H Fujii M Yamamoto T Horie M. Comparison of the effects of efonidipine and amlodipine on aldosterone in patients with hypertension. Hypertens Res (2007) 30:691–7. 10.1291/hypres.30.691 17917316

[109] Burnay MM Python CP Vallotton MB Capponi AM Rossier MF Role of the capacitative calcium influx in the activation of steroidogenesis by angiotensin II in adrenal glomerulosa cells. Endocrinology (1994) 135:751–8. 10.1210/en.135.2.751 8033823

[110] Rossier MF Capponi AM Antagonistes calciques et inhibition de la sécrétion d’aldostérone. Métabol Horm Nutr (2000) IV:101–6.

[111] Li X Yang MS. Effects of T-type calcium channel blockers on renal function and aldosterone in patients with hypertension: a systematic review and meta-analysis. PLoS One (2014) 9:e109834. 10.1371/journal.pone.0109834 25330103 PMC4201480

[112] Rossier MF T channels and steroid biosynthesis: in search of a link with mitochondria. Cell Calcium (2006) 40:155–64. 10.1016/j.ceca.2006.04.020 16759697

[113] Hosey MM Lazdunski M Calcium channels: molecular pharmacology, structure and regulation. J Membr Biol (1988) 104:81–105. 10.1007/BF01870922 2903935

[114] Hescheler J Schulz G G-proteins involved in the calcium channel signalling system. Curr Opin Neurobiol (1993) 3:360–7. 10.1016/0959-4388(93)90129-M 8396476

[115] Lu H-K Fern RJ Luthin D Linden J Liu L-P Cohen CJ Angiotensin II stimulates T-type calcium channel currents *via* activation of a G protein, Gi. Am J Physiol (1996) 271:C1340–9. 8897841 10.1152/ajpcell.1996.271.4.C1340

[116] Lu H-K Fern RJ Nee JJ Barrett PQ Calcium-dependent activation of T-type calcium channels by calmodulin-dependent protein kinase II. Am J Physiol (1994) 267:F183–9. 8048559 10.1152/ajprenal.1994.267.1.F183

[117] Fern RJ Hahm MS Lu H-K Liu L-P Gorelick FS Barrett PQ Calcium/calmodulin-dependent protein kinase II activation and regulation of adrenal glomerulosa calcium signaling. Am J Physiol (1995) 269:F751–60. 8594869 10.1152/ajprenal.1995.269.6.F751

[118] Barrett PQ Lu HK Colbran R Czernik A Pancrazio JJ Stimulation of unitary T-type Ca(2+) channel currents by calmodulin-dependent protein kinase II [In Process Citation]. Am J Physiol Cell Physiol (2000) 279:C1694–703. 11078683 10.1152/ajpcell.2000.279.6.C1694

[119] Rossier MF Burnay MM Maturana AD Capponi AM. Duality of the voltage-dependent calcium influx in adrenal glomerulosa cells. Endocr Res (1998) 24:443–7. 10.3109/07435809809032631 9888523

[120] Maturana AD Burnay MM Capponi AM Vallotton MB Rossier MF. Angiotensin II type 1 receptor activation modulates L- and T-type calcium channel activity through distinct mechanisms in bovine adrenal glomerulosa cells. J Recept Signal Transduct Res (1999) 19:509–20. 10.3109/10799899909036668 10071781

[121] McCarthy RT Isales CM Bollag WB Rasmussen H Barrett PQ. Atrial natriuretic peptide differentially modulates T- and L-type calcium channels. Am J Physiol (1990) 258:F473–8. 2156446 10.1152/ajprenal.1990.258.3.F473

[122] Osipenko ON Varnai P Mike A Spät A Vizi ES. Dopamine blocks T-type calcium channels in cultured rat adrenal glomerulosa cells. Endocrinology (1994) 134:511–4. 10.1210/endo.134.1.7903936 7903936

[123] Drolet P Bilodeau L Chorvátová A Laflamme L Gallo-Payet N Payet MD Inhibition of the T-type calcium current by the dopamine D1 receptor in rat adrenal glomerulosa cells: requirement of the combined action of the G beta gamma protein subunit and cyclic adenosine 3’,5’-monophosphate. Mol Endocrinol (1997) 11:503–14. 10.1210/me.11.4.503 9092802

[124] Lenglet S Louiset E Delarue C Vaudry H Contesse V. Activation of 5-HT(7) receptor in rat glomerulosa cells is associated with an increase in adenylyl cyclase activity and calcium influx through T-type calcium channels. Endocrinology (2002) 143:1748–60. 10.1210/endo.143.5.8817 11956157

[125] Lesouhaitier O Kodjo MK Cartier F Contesse V Yon L Delarue C The effect of the endozepine triakontatetraneuropeptide on corticosteroid secretion by the frog adrenal gland is mediated by activation of adenylyl cyclase and calcium influx through T-type calcium channels. Endocrinology (2000) 141:197–207. 10.1210/en.141.1.197 10614640

[126] Rossier MF Capponi AM Angiotensin II and calcium channels. Vitam Horm (2001) 60:229–84. 10.1016/S0083-6729(00)60021-3 11037626

[127] Hunyady L Rohacs T Bago A Deak F Spät A Dihydropyridine-sensitive initial component of the angiotensin II-induced calcium response in rat adrenal glomerulosa cells. Am J Physiol (1994) 266:C67–72. 7508191 10.1152/ajpcell.1994.266.1.C67

[128] Brauneis U Vassilev PM Quinn SJ Williams GH Tillotson DL Angiotensin II blocks potassium currents in zona glomerulosa cells from rat, bovine and human adrenals. Am J Physiol (1991) 260:E772–9. 2035634 10.1152/ajpendo.1991.260.5.E772

[129] Vassilev PM Kanazirska MV Quinn SJ Tillotson DL Williams GH Potassium channels in adrenal zona glomerulosa cells: I. Am J Physiol (1992) 263:E752–9. 1415696 10.1152/ajpendo.1992.263.4.E752

[130] Kanazirska MV Vassilev PM Quinn SJ Tillotson DL Williams GH Single potassium channels in adrenal zona glomerulosa cells: II. Inhibition by angiotensin II. Am J Physiol (1992) 263:E760–5. 1415697 10.1152/ajpendo.1992.263.4.E760

[131] Chorvátová A Gallo-Payet N Casanova C Payet MD. Modulation of membrane potential and ionic currents by the AT1 and AT2 receptors of angiotensin II. Cell Signal (1996) 8:525–32. 10.1016/S0898-6568(96)00117-9 9115844

[132] Lotshaw DP Characterization of angiotensin II-regulated potassium conductance in rat adrenal glomerulosa cells. J Membr Biol (1997) 156:261–77. 10.1007/s002329900206 9096067

[133] Payet MD Bilodeau L Drolet P Ibarrondo J Guillon G Gallo-Payet N Modulation of a calcium-activated potassium channel by angiotensin II in rat adrenal glomerulosa cells: involvement of a G protein. Mol Endocrinol (1995) 9:935–47. 10.1210/me.9.8.935 7476991

[134] Lobo MV Marusic ET Effect of angiotensin II, ATP, and ionophore A23187 on potassium efflux in adrenal glomerulosa cells. Am J Physiol (1986) 250:E125–30. 3082214 10.1152/ajpendo.1986.250.2.E125

[135] Lobo MV Marusic ET. Angiotensin II causes a dual effect on potassium permeability in adrenal glomerulosa cells. Am J Physiol (1988) 254:E144–9. 3348367 10.1152/ajpendo.1988.254.2.E144

[136] Shepherd RM Fraser R Nichols DJ Kenyon CJ. Efflux of potassium ions in angiotensin II-stimulated bovine adrenocortical cells. J Endocrinol (1991) 128:297–304. 10.1677/joe.0.1280297 2005419

[137] Lobo MV Mendoza RR Marusic ET. sn-1,2 dioctanoylglycerol mimics the effects of angiotensin II on aldosterone production and potassium permeability in isolated bovine glomerulosa cells. J Steroid Biochem (1990) 35:29–33. 10.1016/0022-4731(90)90141-E 2308328

[138] Shepherd RM Fraser R Kenyon CJ Membrane permeability to potassium and the control of aldosterone synthesis: effects of valinomycin and cromakalim in bovine adrenocortical cells. J Mol Endocrinol (1992) 9:165–73. 10.1677/jme.0.0090165 1418387

[139] Lotshaw DP Effects of potassium channel blockers on potassium channels, membrane potential, and aldosterone secretion in rat adrenal zona glomerulosa cells. Endocrinology (1997) 138:4167–75. 10.1210/en.138.10.4167 9322926

[140] Hadjokas NE Goodfriend TL. Inhibition of aldosterone production and angiotensin action by drugs affecting potassium channels. Pharmacol (1991) 43:141–50. 10.1159/000138839 1775517

[141] Chorvátová A Guyot A Ojeda C Rougier O Bilbaut A Activation by angiotensin II of calcium-dependent potasium and chloride currents in zona fasciculata cells of bovine adrenal gland. J Membr Biol (1998) 162:39–50. 10.1007/s002329900340 9516236

[142] Mlinar B Biagi BA Enyeart JJ A novel potassium current inhibited by adrenocorticotropic hormone and angiotensin II in adrenal cortical cells. J Biol Chem (1993) 268:8640–4. 8386167

[143] Wickman K Clapham DE. Ion channel regulation by G proteins. Physiol Rev (1995) 75:865–79. 7480165 10.1152/physrev.1995.75.4.865

[144] Aptel HBC Johnson EIM Vallotton MB Rossier MF Capponi AM. Demonstration of an angiotensin II-induced negative feedback effect on aldosterone synthesis in isolated rat adrenal zona glomerulosa cells. Mol Cell Endocrinol (1996) 119:105–11. 10.1016/0303-7207(96)03805-1 8793859

[145] Louiset E Contesse V Groussin L Cartier D Duparc C Barrande G Expression of serotonin7 receptor and coupling of ectopic receptors to protein kinase A and ionic currents in adrenocorticotropin-independent macronodular adrenal hyperplasia causing Cushing’s syndrome. J Clin Endocrinol Metab (2006) 91:4578–86. 10.1210/jc.2006-0538 16954157

[146] Aguilera G Catt KJ Dopaminergic modulation of aldosterone secretion in the rat. Endocrinology (1984) 114:176–81. 10.1210/endo-114-1-176 6317344

[147] Rossier MF Krause K-H Lew PD Capponi AM Vallotton MB. Control of cytosolic free calcium by intracellular organelles in bovine adrenal glomerulosa cells: effects of sodium and inositol 1,4,5-trisphosphate. J Biol Chem (1987) 262:4053–8. 2435728

[148] Capponi AM Rossier MF Davies E Vallotton MB. Calcium stimulates steroidogenesis in permeabilized bovine adrenal cortical cells. J Biol Chem (1988) 263:16113–7. 2460443

[149] Rossier MF Confinement of intracellular calcium signaling in secretory and steroidogenic cells. Eur J Endocrinol (1997) 137:317–25. 10.1530/eje.0.1370317 9368495

[150] Cherradi N Rossier MF Vallotton MB Capponi AM. Calcium stimulates intramitochondrial cholesterol transfer in bovine adrenal glomerulosa cells. J Biol Chem (1996) 271:25971–5. 10.1074/jbc.271.42.25971 8824233

[151] Cherradi N Rossier MF Vallotton MB Timberg R Friedberg I Orly J Submitochondrial distribution of three key steroidogenic proteins (steroidogenic acute regulatory protein, P450 side-chain cleavage and 3β-hydroxysteroid dehydrogenase isomerase enzymes) upon stimulation by intracellular calcium in adrenal glomerulosa cells. J Biol Chem (1997) 272:7899–907. 9065457 10.1074/jbc.272.12.7899

[152] Brandenburger Y Kennedy ED Python CP Rossier MF Vallotton MB Wollheim CB Possible role for mitochondrial calcium in angiotensin II- and potassium-stimulated steroidogenesis in bovine adrenal glomerulosa cells. Endocrinology (1996) 137:5544–51. 10.1210/endo.137.12.8940382 8940382

[153] Nagai T Sawano A Park ES Miyawaki A. Circularly permuted green fluorescent proteins engineered to sense Ca2+. Proc Natl Acad Sci U S A (2001) 98:3197–202. 10.1073/pnas.051636098 11248055 PMC30630

[154] Lalevee N Resin V Arnaudeau S Demaurex N Rossier MF. Intracellular transport of calcium from plasma membrane to mitochondria in adrenal H295R cells: implication for steroidogenesis. Endocrinology (2003) 144:4575–85. 10.1210/en.2003-0268 12960050

[155] Putney JW Jr. A model for receptor-regulated calcium entry. Cell Calcium (1986) 7:1–12. 10.1016/0143-4160(86)90026-6 2420465

[156] Putney JW Jr Capacitative calcium entry revisited. Cell Calcium (1990) 11:611–24. 10.1016/0143-4160(90)90016-N 1965707

[157] Putney JW Jr “Kissin’ cousins”: intimate plasma membrane-endoplasmic reticulum interactions underlie capacitative calcium entry. Cell (1999) 99:5–8. 10.1016/S0092-8674(00)80056-2 10520988

[158] Gray LS Perez-Reyes E Gomora JC Haverstick DM Shattock M McLatchie L The role of voltage gated T-type Ca2+ channel isoforms in mediating “capacitative” Ca2+ entry in cancer cells. Cell Calcium (2004) 36:489–97. 10.1016/j.ceca.2004.05.001 15488598

[159] Yanagibashi K Papadopoulos V Masaki E Iwaki T Kawamura M Hall PF. Forskolin activates voltage-dependent Ca2+ channels in bovine but not in rat fasciculata cells. Endocrinology (1989) 124:2383–91. 10.1210/endo-124-5-2383 2539978

[160] Yanagibashi K Kawamura M Hall PF. Voltage-dependent Ca2+ channels are involved in regulation of steroid synthesis by bovine but not rat fasciculata cells. Endocrinology (1990) 127:311–8. 10.1210/endo-127-1-311 1694493

